# WRN loss accelerates abnormal adipocyte metabolism in Werner syndrome

**DOI:** 10.1186/s13578-023-01183-4

**Published:** 2024-01-06

**Authors:** Yuyao Tian, Sofie Lautrup, Patrick Wai Nok Law, Ngoc-Duy Dinh, Evandro Fei Fang, Wai-Yee Chan

**Affiliations:** 1 Faculty of Medicine, School of Biomedical Sciences, The Chinese University of Hong Kong, Shatin, N.T. Hong Kong SAR; 2 Department of Biomedical Engineering, The Chinese University of Hong Kong, Shatin, N.T. Hong Kong SAR; 3 Department of Clinical Molecular Biology, University of Oslo and Akershus University Hospital, 1478 Lørenskog, Norway; 4 Hong Kong Branch CAS Center of Excellence for Animal Evolution and Genetics, The Chinese University of Hong Kong, Shatin, N.T. Hong Kong SAR; 5 CUHK-SDU University Joint Laboratory on Reproductive Genetics, The Chinese University of Hong Kong, Shatin, N.T. Hong Kong SAR; 6 MOE Key Laboratory of Regenerative Medicine (CUHK-Jinan University), The Chinese University of Hong Kong, Shatin, N.T. Hong Kong SAR

**Keywords:** Werner syndrome, WRN, Abnormal metabolism, SMARCA5

## Abstract

**Background:**

Metabolic dysfunction is one of the main symptoms of Werner syndrome (WS); however, the underlying mechanisms remain unclear. Here, we report that loss of *WRN* accelerates adipogenesis at an early stage both in vitro (stem cells) and in vivo (zebrafish). Moreover, *WRN* depletion causes a transient upregulation of late-stage of adipocyte-specific genes at an early stage.

**Methods:**

In an in vivo study, we generated *wrn*^*−/−*^ mutant zebrafish and performed histological stain and Oil Red O staining to assess the fat metabolism. In an in vitro study, we used RNA-seq and ATAC-seq to profile the transcriptional features and chromatin accessibility in *WRN* depleted adipocytes. Moreover, we performed ChIP-seq to further study the regulatory mechanisms of metabolic dysfunction in WS.

**Results:**

Our findings show that mechanistically WRN deficiency causes SMARCA5 upregulation. SMARCA5 is crucial in chromatin remodeling and gene regulation. Additionally, rescuing WRN could normalize SMARCA5 expression and adipocyte differentiation. Moreover, we find that nicotinamide riboside (NR) supplementation restores adipocyte metabolism in both stem cells and zebrafish models.

**Conclusions:**

Our findings unravel a new mechanism for the influence of WRN in the early stage of adipogenesis and provide a possible treatment for metabolic dysfunction in WS. These data provide promising insights into potential therapeutics for ageing and ageing-related diseases.

**Supplementary Information:**

The online version contains supplementary material available at 10.1186/s13578-023-01183-4.

## Introduction

Werner syndrome (WS) is a rare autosomal recessive genetic disease marked by premature aging [1]. Metabolic dysregulation is a prominent biochemical characteristic of WS, manifested as loss of subcutaneous fat, dyslipidemia, diabetes, and aberrant glucose metabolism [1–4]. However, the underlying mechanism for these dysfunctions remain largely unknown. WS is caused by mutations in the *WRN* gene, encoding WRN protein, a member of the RecQ helicase family. WRN protein has exonuclease and strand annealing functions [5]. WRN is an important factor in chromatin stability, and patients with WS and WRN mutant models exhibit increased sensitivity to DNA damage, underscoring the role of WRN in DNA repair [6].

Adipocyte tissue is essential for maintaining metabolic homeostasis and fitness throughout life [7]. Adipogenesis requires a sophisticated and fine-tuned differentiation program at different stages that is regulated by certain transcription factors and regulators [8–10]. White, brown, and beige adipocytes are the three basic types of adipocytes [11]. White adipose tissue (WAT), mainly consisting of subcutaneous and visceral fat, stores energy, whereas brown adipose tissue (BAT) is used to produce heat [11, 12] and beige adipocyte tissue is able to generate heat during cold environment [12]. Aging has been shown to accelerate adipogenesis at the expense of osteogenesis in bone marrow-derived mesenchymal stroma/stem cells (mMSC) [13]. It has been demonstrated that aging activated the commitment to the adipocyte lineage, and the increased expression of *PPARγ2* accompanied with ageing process [13]. Thus, dissecting the mechanisms of adipogenesis is likely to be relevant to understanding the pathology of metabolic abnormalities and impairments in aging and aging-related diseases. However, the mechanism by which WRN affects adipogenesis during WS remains unclear.

During cellular development, a series of systematic changes in gene expression are required, and adipocyte development is no exception [14]. Dynamic chromatin alternations, such as changes in chromatin accessibility, conformation, and other modifications, are integral in cell differentiation as well [15]. Mammalian cells typically have active euchromatin and idle heterochromatin. When chromatin is in the active state, some areas containing DNA regulatory elements in the chromatin will become open, enabling binding of transcription factors, which promotes gene expression [16, 17]. The development of assay for transposase-accessible chromatin using sequencing (ATAC-seq) technique has enabled a new way to study the influence of chromatin on gene expression [18, 19]. Knowledge about the network of interactions governing chromatin accessibility and gene regulation during adipocyte differentiation in WS is currently lacking, and would benefit from approaches such as ATAC-seq.

Nicotinamide adenine dinucleotide (NAD^+^) is essential for metabolism and has a role in regulating various metabolic pathways [20]. NAD^+^ homeostasis is necessary for maintaining fitness of a variety of metabolic tissues, such as fat, liver and intestines [21, 22], that the level of NAD^+^ is affected by multiple factors such as ageing, disturbed metabolic status, and disruption of the circadian rhythm [20, 22]. In line with this mechanism, supplementation with nicotinamide mononucleotide (NMN), which is an intermediate in NAD^+^ biosynthesis, can normalize metabolic derangements in adipocyte-specific nicotinamide phosphoribosyl transferase (NAMPT) mutant mice [23]. Furthermore, a recent study showed that NAD^+^ supplements nicotinamide riboside (NR) and NMN restored low NAD^+^ levels and protect against metabolic dysfunction in rodent models, underlying that NAD^+^ supplementation could be a promising therapeutic to safeguard metabolic health and ameliorate aging [24].

Here, we demonstrated that loss of *WRN* led to accelerated adipogenesis in WS. Moreover, we found that loss of *WRN* caused the expression of late adipocyte-associated circadian markers to exhibit a transient peak at an early stage. Using RNA-seq and ATAC-seq analyses, we revealed that WRN depletion increased chromatin accessibility during adipogenesis. Furthermore, we noted that loss of WRN led to aberrant upregulation of *SMARCA5*, which was responsible for accelerated adipogenesis. Altogether, our findings describe an unsuspected role of SMARCA5 in contributing to metabolic dysfunction, which may provide a promising therapeutic target for WS.

## Methods

### Adipocytes differentiation of hMSCs

Human mesenchymal stem cells were kindly given by Prof. Chan Hon Fai Vivas (School of Biomedical Sciences, The Chinese University of Hong Kong) and differentiated into adipocytes following the manufacturer’s instructions (Thermo Fisher, A1007001). The differentiation medium was changed every 2–3 days.

### Brown and white adipocytes differentiation

Brown and white adipocytes differentiation followed previous optimized protocol [25]. Generally, the undifferentiated cells were maintained in DMEM/F12 medium with 20% KSR with floating cultivation to form embryoid bodies (EB) using low attachment six-wells. 10^−6^M retinoic acid (RA) was added from day 3 to day 5 for white adipocytes only. After 10 days culture, EBs were seeded onto gelatin-coated plates in DMEM/F12 medium with 20% KSR. In the meanwhile, the cells were induced for adipocyte differentiation using a cocktail with DMEM/F12 medium with 10% KSR (0.5 mM IBMX, 0.25 µM Dex, 0.2 nM T3, 1 µg/ml insulin, 1 µM Rosiglitazone). The differentiation medium was renewed every 3 days till day 30.

### WRN deletion in human MSCs

The Cas9 target sequences for *WRN* deletion were designed as previously reported [26]. Guide sequence of the sgRNA were incorporated into pSpCas9(BB)-2 A-GFP vector (Addgene, 48138) including the *Cas9* and green fluorescent protein (*GFP*) genes. hMSCs were cultured in 6-well plates and transfected with constructs containing Cas9 and target sequence using FuGENE® transfection reagent (Promega, E5911, USA). The cells were sorted by GFP-positive cells after 2 days. The correct cell lines were identified by DNA sequencing.

### Flow cytometry assay

The cells were harvested and cell number was adjusted to a concentration of 1–2 × 10^5^ cells/ml in ice cold FACS buffer. The cell suspension was aliquoted to 200 µl per tube and analyzed on a flow cytometer BD LSR Fortessa cell analyzer using a 488-nm laser.

### Oil Red O staining

Oil Red O staining was performed according to a previous study [27]. Briefly, following anesthesia with MS-222 (200 ppm), zebrafish were fixed in 4% PFA for 12 h at 4 ℃ and washed three times with PBS. Fish were subsequently pre-incubated in 60% isopropanol for 30 min and dyed with fresh 0.3% Oil Red O for 3 h. Samples were prepared for microscopic observation after three washes with 10% isopropanol.

### Histological examination

At the end of the treatments, the *wrn*^*−/−*^ mutant zebrafish and healthy control siblings were subjected to histological examination. Adipose tissue samples were preserved in freshly prepared fixative 4% paraformaldehyde (PFA). The next day, the samples were subjected to standard histological procedures, including paraffin embedding, sectioning, and staining with hematoxylin and eosin (HE). Images were taken with a Leica SP8 confocal microscope.

### RNA extraction, cDNA synthesis and quantitative real-time polymerase chain reaction

Total RNA was extracted using Direct-zol RNA Microprep kit (R2062, Zymo Research, USA) and TRIzol reagent (Invitrogen, USA) following the manufacturer’s instructions. Reverse transcription was performed using a MasterMix kit (Takara, Japan), according to the manufacturer’s instructions. Quantitative polymerase chain reaction (qRT-PCR) was performed using a Universal SYBR Green MasterMix (Takara, Japan) on a QuantStudio 7 Flex real-time PCR system. All the primers sequences were provided in the Additional file 1.

### RNA-seq preparation and analysis

RNA-seq for WT and WRN KO adipocytes on days 1, 5, and 21 were performed. The samples were frozen in liquid nitrogen and then sent to Novogene (Tianjin, China), where the library was prepared, and sequencing was performed with Novaseq6000 platform. Three biological replicates were performed.

DESeq2 was used to identify Differentially expressed genes (DEG). Significance was set at *padj* < 0.05. PCA and KEGG plots were generated using ggplot2 in RStudio (version 2023.6.1.524).

### ATAC-seq preparation and analysis

The ATAC-seq was performed following the manufacturer’s instructions (Active Motif kit, 53150, USA) with minor modifications. Generally, 1 × 10^6^ wild-type or WRN-KO adipocytes were collected on days 1 and 5. Each sample was lysed in the ATAC-seq lysis buffer. Next the samples were processed for the transposase reaction, library generation and purification. The samples were sent to Novogene (Tianjin, China) and sequenced. Two biological replicates were used for ATAC-seq.

ATAC-seq reads were aligned to the human genome hg19 using Bowtie2 (bowtie2-p 8-X 2000-no-mixed–no-discordant) [28]. ATAC-seq de novo motif discovery was conducted using HOMER (http://www.homer.ucsd.edu).

### Chromatin immunoprecipitation sequencing (ChIP-seq) and ChIP-qPCR

The ChIP-seq or ChIP-qPCR samples were prepared following the manufacturer’s instruction (Active Motif, 53035, USA). Approximately 1.5 × 10^7^ wild-type or WRN-KO adipocytes were collected on days 1 and 5. Each sample was fixed with the fixation solution. After 10 min fixation, it was terminated by glycine stop-fix solution. The chromatin was sheared by enzymatic shearing (Active Motif, 53009, USA) with minor modifications. The sheared chromatin was processed for chromatin immunoprecipitation and purification. 10% of chromatin was saved as input control. The 15 µg WRN antibody (Sigma, W0393) was used for the immunoprecipitation. The ChIP-qPCR primers were provided in Additional file 1: Table S1.

### Zebrafish maintenance

All zebrafish husbandry and maintenance procedures were approved by the Animal Ethics Committee of the Chinese University of Hong Kong. The F1 mutant, *wrn*^sa34829^ (C > T), which was generated using an ENU method, were purchased from the Zebrafish Information Network (ZFIN, zfin.org).

### Rescue experiments with NR treatment

NR was given as provided by Prof. Evandro Fang Fei (Department of Clinical Molecular Biology, University of Oslo and Akershus University Hospital) prepared as a 10 mM stock solution. A working solution of NR at 10 µM was prepared to treat the WRN cells and at 100 µM to treat the *wrn*^*−/−*^ mutant zebrafish. The embryos were incubated in the system water with or without NR and collected at different time points.

### Data collection and statistical analysis

Two-tailed unpaired t-tests were used for biostatistics. All data were presented as mean ± SD as indicated, with *P < 0.05 considered statistically significant.

## Results

### WRN deficiency accelerates adipocyte metabolism

**Fig. 1:**
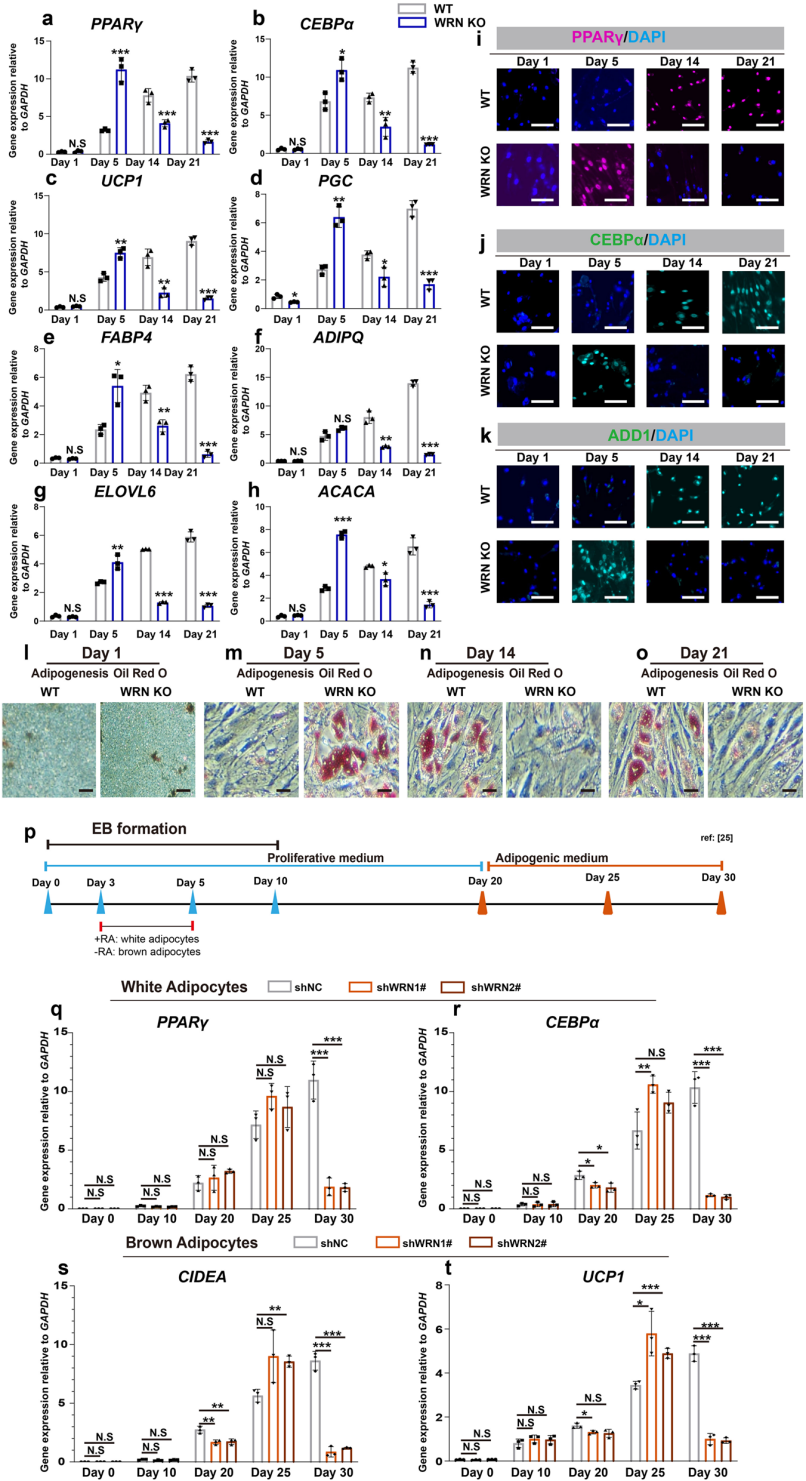
WRN deficiency accelerates adipocyte metabolism. **a**–**h** qRT-PCR analysis of selected adipogenic markers *PPARγ* (**a**), *CEBPα* (**b**), *UCP1* (**c**), *PGC* (**d**), *FABP4* (**e**), *ADIPQ* (**f**), *ELOVL6* (**g**), and *ACACA* (**h**) in hMSCs (N = 3 biological replicates). **i**–**k** Representative immunofluorescent images of PPARγ (**i**), CEBPα (**j**), and ADD1 (**k**) (N = 3 biological replicates). Scale bar = 20 μm. **l**–**o** Representative Oil Red O staining images during adipogenesis (N = 3 biological replicates). **p** Illustration of white and brown adipocytes differentiation following previous report [25]. **q**, **r** qRT-PCR analysis of selected white adipogenic markers *PPARγ* (**q**) and *CEBPα* (**r**) in hESCs (N = 3 biological replicates). **s**, **t** qRT-PCR analysis of selected brown adipogenic markers *CIDEA* (**s**), and *UCP1* (**t**) in hESCs (N = 3 biological replicates). Data are presented as the mean ± S.D. Statistical analysis was performed using two-tailed unpaired Student’s t-test. *P < 0.05, **P < 0.01, ***P < 0.001

To test the role of WRN in the adipocyte developmental process, we firstly used human mesenchymal stem cells (hMSCs). Mesenchymal stem cells (MSCs) can be committed to adipocyte lineage, making them a good model for studying adipogenesis [14]. We collected samples at different time points during adipogenesis (day 1, day 5, day 14, and day 21) for analysis. We generated *WRN* knock-out hMSCs (indicated as WRN KO hereafter) using the CRISPR/Cas-9 technique and WRN guide RNA design following a previous report [26]. We used fluorescence-activated cell sorting (FACS) to pick out CRISPR/Cas-9 positive cells and confirmed the knockout efficiency using qRT-PCR (Additional file 2: Fig. S1a). Next, we measured the mRNA expression of selected adipocyte-associated and lipocyte-associated genes (Fig. 1a–h), including peroxisome proliferator-activated receptor gamma (*PPARγ*, a), CCAAT enhancer binding protein alpha (*CEBPα*, b), uncoupling protein 1 (*UCP1*, c), Progastricsin (*PGC*, d), fatty acid binding protein 4 (*FABP4*, e), Adiponectin (*ADIPQ*, f), ELOVL fatty acid elongase 6 (*ELOVL6*, g), and Acetyl-coA carboxylase alpha (*ACACA*, h). When compared to the expression level in wildtype (indicated as WT hereafter), we noted that they were transiently upregulated in WRN KO cells, and with the most notably difference on day 5 (Fig. 1a–h). The mRNA expression of the above-mentioned genes decreased dramatically in the WRN KO groups at the late adipogenic stage, while they gradually increased in the WT groups (Fig. 1a–h). Immunofluorescent staining of PPARγ (i), CEBPα (j), and adipocyte determination and differentiation-dependent factor 1 (ADD1) (k) validated the qRT-PCR findings, and showed more abundant signals in WRN KO adipocytes on day 5 (Fig. 1i–k). In line with these findings, Oil Red O staining showed more accumulation of lipid droplets in WRN KO cells on day 5 compared to that seen in WT cells, and decreased in subsequent days (Fig. 1l–o).

Next, to examine whether the role of WRN is consistent between different adipocyte types, we differentiated human embryonic stem cells (hESCs) into either white or brown adipocytes, as previously reported [25]. We generated two WRN knock-down (indicated as WRN-KD hereafter) cells (shWRN1# and shWRN2#) following a previous report [29]. The control cells (indicated as shNC hereafter) and two WRN-KD cell lines were differentiated into embryonic bodies (EBs) to induce the formation of three germ layers and were cultured for 10 days. The isolation of both white and brown adipocytes was mainly based on early treatment of EBs with or without retinoic acid (RA), followed by differentiation in adipocyte medium for 20 days (Fig. 1p). The expression of adipocyte-related genes showed no pronounced difference on day 10, indicating that loss of WRN did not affect the pluripotent stage (Fig. 1q–t). On day 25, the expression of representative adipocyte markers was enhanced in shWRN1# and 2# groups compared to that in the shNC groups, indicating that loss of *WRN* speeds up the ability of adipocyte differentiation (Fig. 1q–t). Interestingly, we noted that WRN did not show pronounced difference between white adipocytes and brown adipocytes. Altogether, these results demonstrate that depletion of WRN accelerates adipocyte metabolism at an early stage, but inhibits it at a late stage.

### Late-stage adipogenic genes express earlier abnormally

**Fig. 2:**
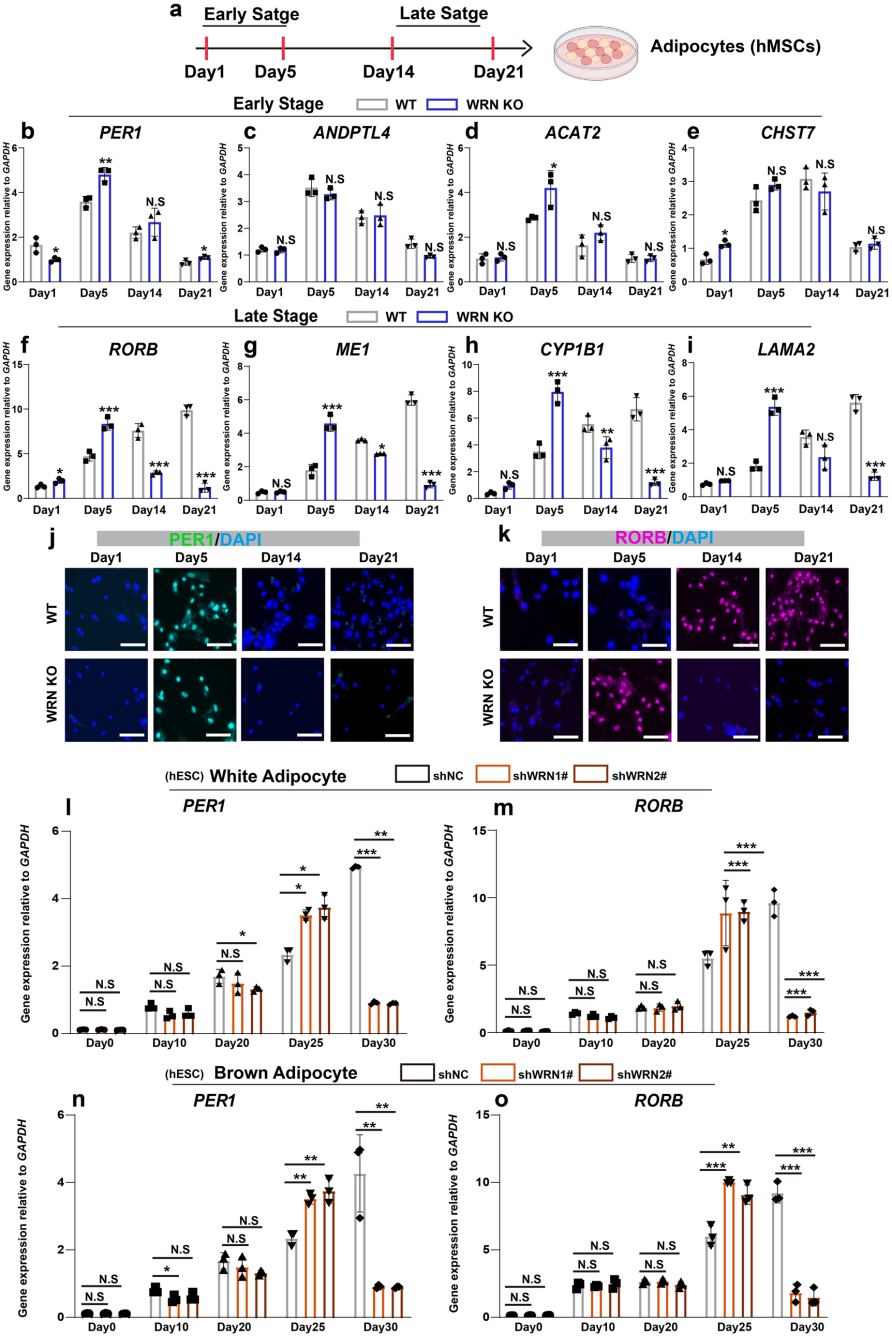
Late-stage adipogenic genes express earlier abnormally. **a** Illustration of the early stage and late stage during adipogenesis. **b**–**e** qRT-PCR analysis of early circadian genes *PER1* (**b**), *ANDPTL4* (**c**), *ACAT2* (**d**), and *CHST7* (**e**) in hMSCs (N = 3 biological replicates). **f**–**i** qRT-PCR analysis of late circadian genes RORB (**f**), ME 1 (**g**), CYP1B1 (**h**), and LAMA 2 (**i**) in hMSCs (N = 3 biological replicates). **j**, **k** Representative immunostaining images of PER1 (**j**) and RORB (**k**). **l**, **m** qRT-PCR analysis of *PER1* (**l**) and *RORB* (**m**) in hESCs-derived white adipocytes (N = 3 biological replicates). **n**, **o** qRT-PCR analysis of *PER1* (**l**) and *RORB* (**m**) in hESCs-derived brown adipocytes (N = 3 biological replicates). Data are presented as the mean ± S.D. Statistical analysis was performed using two-tailed unpaired Student’s t-test. *P < 0.05, **P < 0.01, ***P < 0.001

We next investigated the role of WRN at different stage-specific time points during adipogenesis. We followed a previous report that defined adipocyte differentiation as “early stage” and “late stage” using hMSCs (Fig. 2a) [30]. It has been reported that certain adipocyte differentiation genes only expressed at the early stage (days 1 and 5) or late stage (days 14 and 21) [30]. We firstly examined these early-stage differentiation markers (Fig. 2b–e) with qRT-PCR: period circadian regulator 1 (*PER1*), angiopoietin-like 4 (*ANDPTL4*), acetyl-CoA acetyltransferase-2 (*ACAT2*), and carbohydrate sulfotransferase 7 (*CHST7*). The results showed no significant differences in expression of early-stage adipocyte differentiation markers between the WT and WRN KO adipocytes. Next, late-stage adipocyte differentiation markers, such as retinoic acid-related orphan receptor B (*RORB*), malic enzyme (*ME1*), cytochrome P450 family 1 subfamily member 1 (*CYP1B1*), and laminin subunit alpha 2 (*LAMA2*) were examined (Fig. 2f–i). The expression of the late-stage adipocyte differentiation markers exhibited a transient peak in WRN KO adipocytes compared to their expression in the WT cells on day 5. The qPCR findings were validated by immunostaining of PER1 and RORB (Fig. 2j, k).

We confirmed our findings of the role of WRN on early and late stages of adipogenesis, in both white adipocytes and brown adipocytes using the hESCs model. In this model, mesenchymal progenitor stage begins from day 20, so day 25 is therefore considered as the early stage of adipogenesis. We examined the mRNA expression of *PER1* and *RORB* in white adipocytes and brown adipocytes separately. As Fig. 2l–o shown, the expression of *RORB* increased significantly in the early stage and there was no difference between white and brown adipocytes. Taken together, these results indicate that the late-stage of adipocyte development events happened in advance due to the loss of WRN activity.

### The *wrn*^−/−^ mutant zebrafish shows adipocyte prematurity

**Fig. 3:**
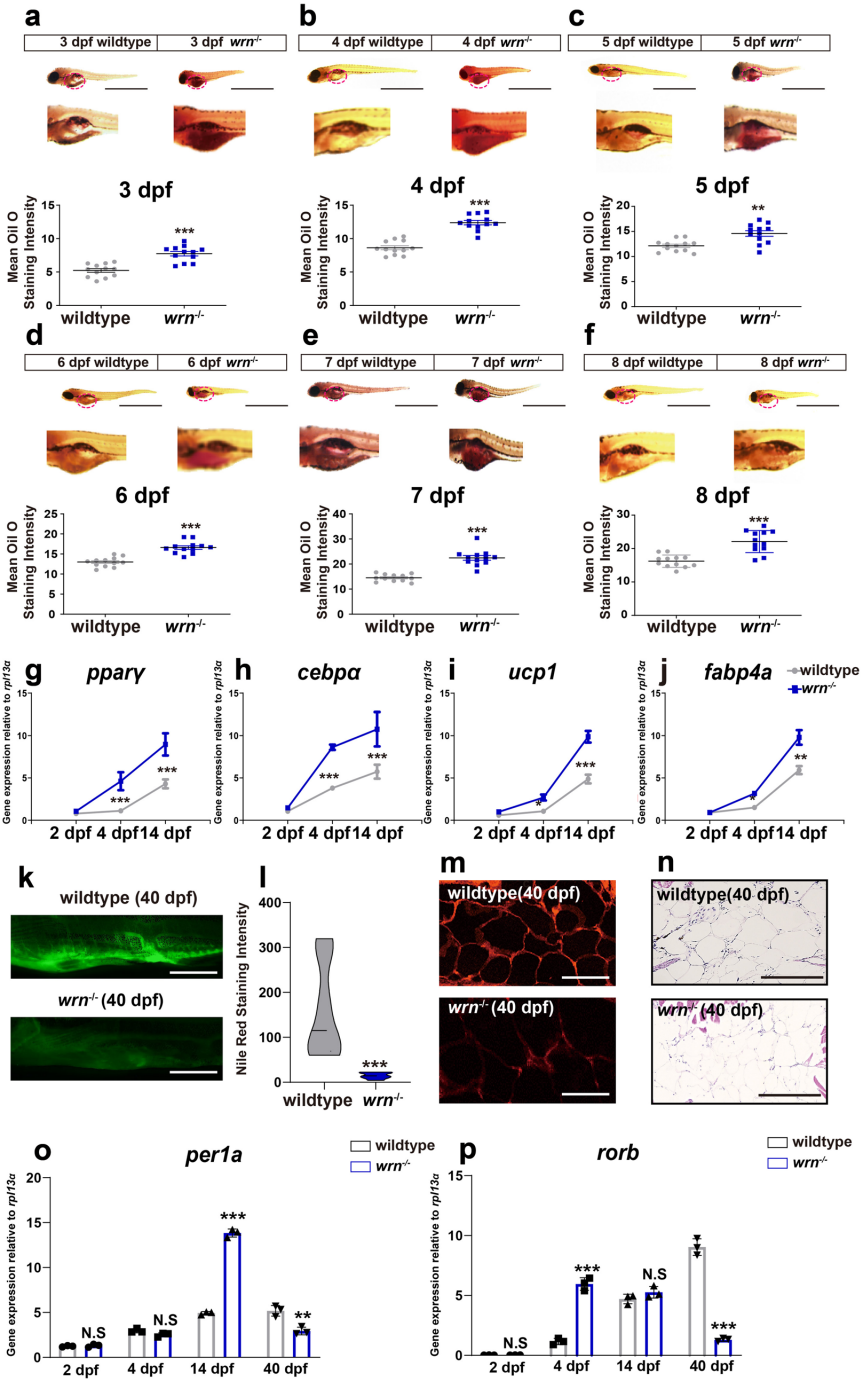
The *wrn*^***−/− ***^mutant zebrafish shows adipocyte prematurity. **a**–**f** Representative images of Oil Red O staining from 3 dpf to 8 dpf (N = 3 biological replicates). Scale bar = 100 μm. **g**–**j** qRT-PCR analysis of selected adipogenic markers *pparγ* (**g**), *cebpα* (**h**), *ucp1* (**i**), and *fabp4* (**j**) at 2 dpf, 4 dpf, and 14 dpf (N = 3 biological replicates). **k** Representative images of Nile Red staining on 40 dpf (N = 3 biological replicates). Scale bar = 100 μm. **l** Violin graph of NileRed staining intensity analysis on 40 dpf (N = 3 biological replicates). **m** Representative images of Oil Red O staining sections (N = 3 biological replicates). Scale bar = 50 μm. **n** Representative images of Hematoxylin and Eosin (H&E) sections (N = 3 biological replicates). Scale bar = 50 μm. **o**, **p** qRT-PCR analysis of *per1a* (**o**) and *rorb* (**p**) (N = 3 biological replicates). Data are presented as the mean ± S.D. Statistical analysis was performed using two-tailed unpaired Student’s t-test. *P < 0.05, **P < 0.01, ***P < 0.001

To investigate the physiological role of WRN in fat development, we used zebrafish, an attractive model for studying adipocyte differentiation due to the high similarity in adipose biology, lipid metabolism and metabolic homeostasis between humans and zebrafish [31, 32]. The *wrn*^*−/−*^ mutant zebrafish were generated as described previously [29]. We performed whole-mount Oil Red O staining of the zebrafish, and *wrn*^*−/−*^ mutant zebrafish exhibited elevated Oil Red O staining intensity compared to wildtype from 3 days post fertilization (dpf) to 8 dpf (Fig. 3a–f). Additionally, the mRNA expression of the adipocyte-specific genes *pparγ*, *cebpα*, *ucp1*, and *fabp4a* was significantly upregulated in *wrn*^*−/−*^ mutant zebrafish at an early stage (Fig. 3g–j).

We further evaluated lipid accumulation using Nile Red staining at 40 dpf, comparing *wrn*^*−/−*^ mutant zebrafish and wildtype. We showed that the signal intensity of Nile Red was significantly lower in *wrn*^*−/−*^ zebrafish compared to wildtype (Fig. 3k, l). We also performed Oil Red O (Fig. 3m) and HE (Fig. 3n) sections staining and noted decreased fat accumulation in *wrn*^*−/−*^ mutant zebrafish. This is in line with other reports showing that WS patients exhibit loss of subcutaneous fat in adulthood [33].

To confirm our in vitro findings, we examined the expression of the early-stage and late-stage adipogenic markers during zebrafish development. As show in Fig. 3o, p, the expression of *per1a* increased at 14 dpf then declined significantly at 40 dpf in *wrn*^*−/−*^ mutant zebrafish. The expression of *rorb* increased at 4 dpf, stayed the same at 14 dpf, but decreased at 40 dpf in *wrn*^*−/−*^ mutant zebrafish. Taken together, these data indicate that loss of *wrn* caused adipocyte premature in zebrafish development.

### Stage-specific gene regulatory pattern during adipocyte differentiation

**Fig. 4:**
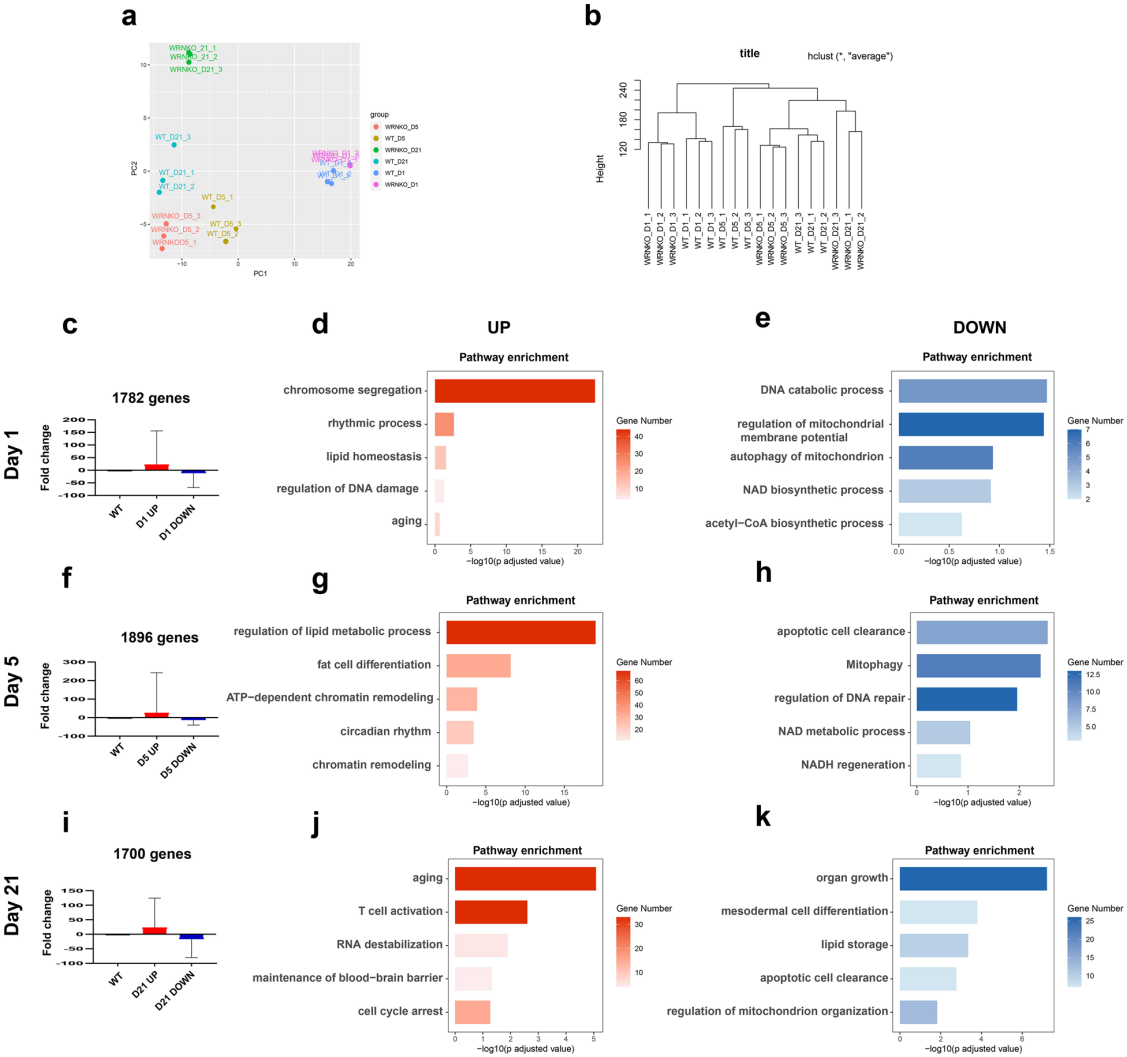
Stage-specific gene regulatory pattern during adipocyte differentiation. **a** Principal component (PCA) analysis of the different replica of the samples (N = 3 biological replicates). **b** Hierarchical clustering (HLC) of the different replica of the samples. **c**, **f**, **i** Fold change of the genes classified as UP and DOWN on day 1 (**c**), day 5 (**f**), and day 21 (**i**). **d**, **g**, **j** Representative Up-regulation KEGG enrichment pathways on day 1 (**d**), day 5 (**g**), and day 21 (**j**). **e**, **h**, **k** Representative down-regulation KEGG enrichment pathways on day 1 (**e**), day 5 (**h**), and day 21 (**k**)

To unmask the stage-specific molecular mechanisms underlying the metabolic dysfunction and transient accelerated adipogenesis driven by *WRN* depletion, we performed transcriptome sequencing (RNA-seq) analysis on days 1, 5, and 21 of adipogenesis, comparing WT and WRN KO adipocytes. Quality validation of raw reads was performed using FastQC (Available online at: http://www.bioinformatics.babraham.ac.uk/projects/fastqc/) [34]. Mean reads quality score was > 40 (Additional file 2: Fig. S2a). Per sequence quality score was > 35 (Additional file 2: Fig. S2b). And we examined the reproducibility of replicates by Pearson’s correlation (Additional file 2: Fig. S2c). Principal component analysis (PCA) and hierarchical clustering (HCL) analysis indicated a good quality of sequencing, manifested as a significant difference in the transcripts of the differential expression genes (DEGs) between the WT and WRN KO cells at three different time points (Fig. 4a, b).

To further analyze the specific gene regulatory patterns at each timepoint during adipogenesis, we applied the Kyoto Encyclopedia of Genes and Genomes (KEGG) pathway analysis and classified the genes according to up and down regulated sequentially. On day 1 of adipogenesis, 1782 genes were differentially expressed in WRN KO versus WT. Representative up-regulated KEGG in WRN KO adipocytes were related to chromosome segregation, rhythmic processes, and lipid homeostasis. Additionally, NAD^+^ biosynthesis, autophagy of mitochondria, and mitochondrial membrane potential activities declined in WRN KO adipocytes (Fig. 4c–e).

On day 5 of adipogenesis, 1896 genes were differentially expressed. Fat cell differentiation and lipid metabolic processes were upregulated in WRN adipocytes in the KEGG pathway analysis, which is consistent with our previous in vivo and in vitro findings (Figs. 1 and 3). Interestingly, chromatin remodeling and circadian rhythms were upregulated as well. Apoptotic cell clearance, NAD^+^ metabolic process, and regulation of DNA repair were found in the downregulated KEGG groups in WRN KO adipocytes (Fig. 4f–h). On day 21 of adipogenesis, 1700 genes significantly changed. We noted that in WRN KO adipocytes, aging and cell cycle arrest related pathways were upregulated, while organ growth and lipid storage were downregulated (Fig. 4i–k). Taken together, these results could be informative to help us better understand the stage-specific regulatory mechanisms of abnormal adipocyte metabolism in WS.

### ATAC-seq profiling reflects adipocytes-related chromatin accessibility changes

The RNA-seq results indicate that adipocyte metabolism and chromatin regulation are intertwined. Recent work has revealed that alterations in the chromatin landscape and resultant modifications in gene expression patterns occurred in aging cells and tissues [35, 36]. Chromatin accessibility is tightly linked to the transcriptional output. In order to understand how chromatin accessibility is affected in the accelerated aging-disease WS, we therefore measured global chromatin accessibility using an assay for transposase-accessible chromatin sequencing (ATAC-seq) on days 1 and 5 of adipogenesis of WT and WRN KO adipocytes.

**Fig. 5:**
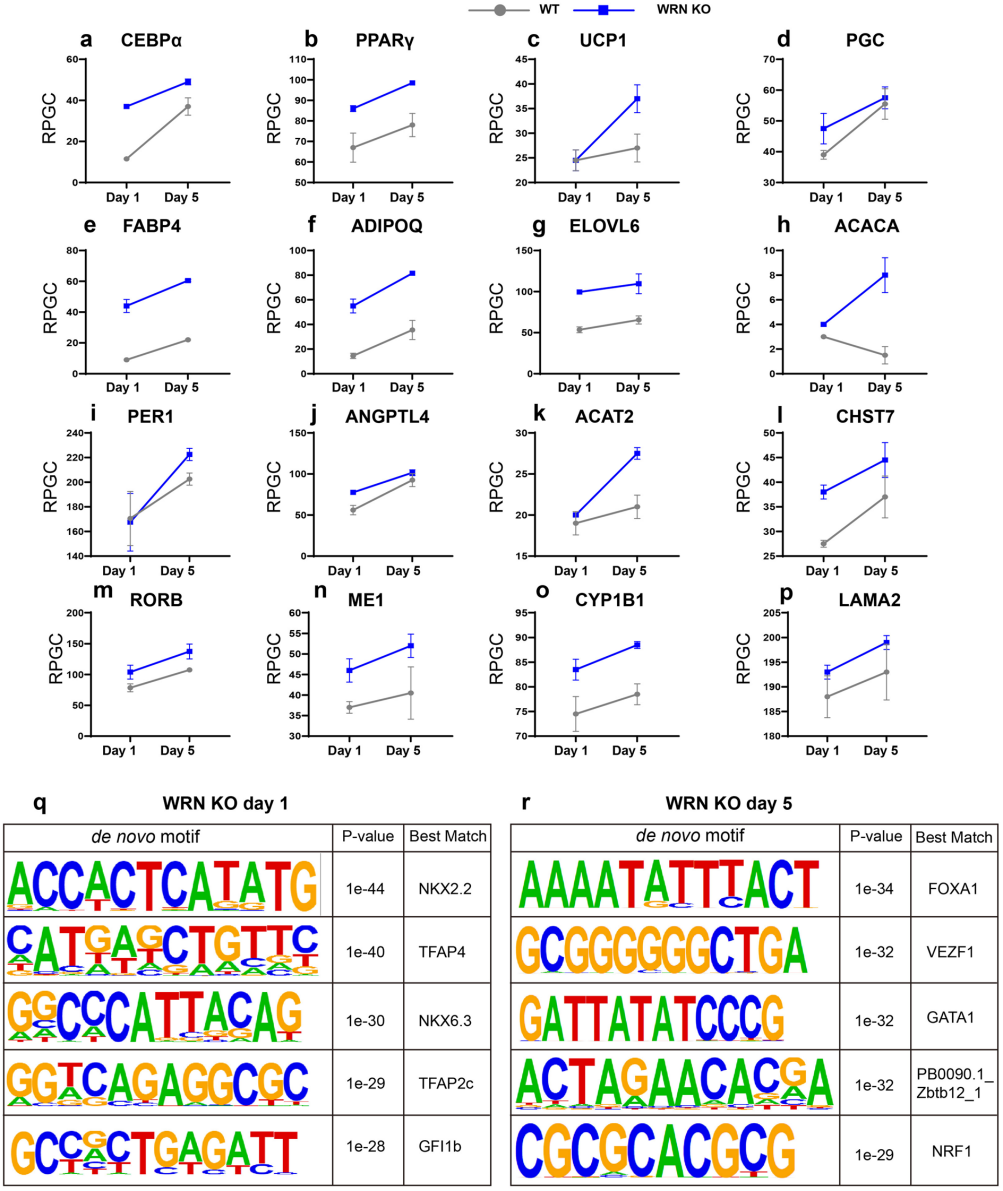
ATAC-seq profiling reflects adipocyte-related chromatin accessibility changes. **a**–**p** Dynamic changes in chromatin accessibility of selected adipogenic associated genes of reads per genome coverage (RPGC) CEBPα (**a**), PPARγ (**b**), UCP1 (**c**), PGC (**d**), FABP4 (**e**), ADIPQ (**f**), ELVOL 6 (**g**), ACACA (**h**), PER 1 (**i**), ANDPTL 4 (**j**), ACAT 2 (**k**), CHST 7 (**l**), RORB (**m**), ME 1 (**n**), CYP1B1 (**o**) and LAMA 2 (**p**). **q**, **r** De novo motif analysis of WRN KO adipocytes on days 1 and 5

Chromatin accessibility of critical genes governing adipocyte differentiation and function was evaluated using reads per genome coverage (RPGC) (Fig. 5a–p). Interestingly, the RPGC analysis indicated that CEBPα (a), PPARγ (b), UCP1 (c), PGC (d), FABP4 (e), ADIPQ (f), ELOVL 6 (g), ACACA (h), PER 1 (i), ANDPTL 4 (j), ACAT 2 (k), CHST 7 (l), RORB (m), ME 1 (n), CYP1B1 (o), and LAMA 2 (p) exhibited open accessibility at different levels on day 5 in the WRN KO cells.

Additionally, we used Homer software (http://www.homer.ucsd.edu) to analyze and identify a set of enriched transcription motifs of WRN mutant adipocytes on days 1 and 5. The top five transcription factors (TFs) motifs on day 1 were NKX2.2, TFAP4, NKX6.3, TFAP2c, and GFI1b (Fig. 5q). Similarly, the top five TF motifs on day 5 were FOXA1, VEZF1, GATA1, PB0090.1_Zbtb12_1, and NRF1 (Fig. 5r).

Taken together, our ATAC-seq data suggest that WRN deficiency leads to chromatin accessibility changes that cause abnormal transcriptional alterations on days 1 and 5 of adipogenesis.

### Hyperactive SMARCA5 causes the adipocyte prematurity in WS

We further studied the molecular mechanisms of adipocyte development in WS. To this end, WRN-ChIP-seq was performed on days 1 and 5 of adipogenesis between WT and WRN KO adipocytes. We overlapped three high-throughput sequencing analysis results (RNA-seq, ATAC-seq, and ChIP-seq) and found that nine genes were upregulated and fourteen downregulated on day 1 of adipogenesis (Additional file 2: Fig. S5a, b). Similarly, eight genes were upregulated, and three genes were downregulated on day 5 (Additional file 2: Fig. 5c, d). One of the genes with increased expression on days 1 and 5 of adipogenesis was the SWI/SNF-related matrix-associated actin-dependent regulator of chromatin subfamily A member 5 *SMARCA5* (also known as *SNF2H*) (Additional file 2: Fig. S5e, f). SMARCA5 is a chromatin remodeler and belongs to SWI/SNF family [37]. Chromatin remodeling SWI/SNF family is necessary for transcriptional regulation [38], cell development, and controls chromatin accessibility for gene expression [39, 40].

**Fig. 6:**
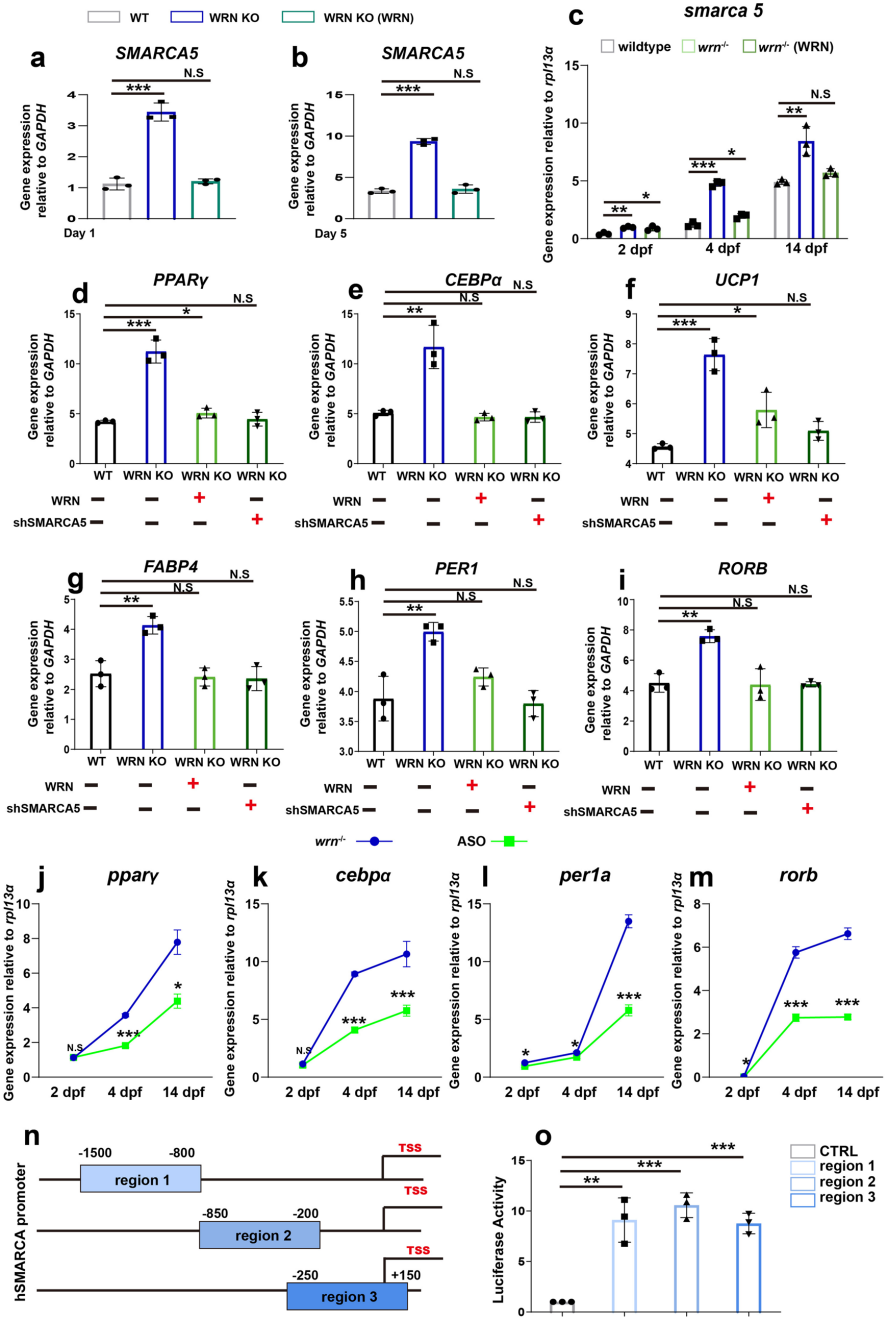
Hyperactive SMARCA5 causes the adipocyte prematurity in WS. **a**, **b** qRT-PCR analysis of *SMARCA5* expression on days 1 (**a**) and 5 (**b**) among WT, WRN KO, and WRN overexpression adipocytes (WRN KO (WRN)) (N = 3 biological replicates). **b** qRT-PCR analysis of *smarca5* expression at 2 dpf, 4 dpf, and 14 dpf among wildtype, *wrn*^*−/−*^ mutant zebrafish, and wrn overexpression zebrafish (*wrn*^*−/−*^ (WRN)) (N = 3 biological replicates). **d**–**g** qRT-PCR analysis of *PPARγ*, *CEBPα*, *UCP1*, and *FABP4* on day 5 among WT, WRN KO, and WRN overexpression or SMARCA5 knock-down adipocytes. (N = 3 biological replicates). **h**, **i** qRT-PCR analysis of *PER1* and *RORB* on day 5 among WT, WRN KO, and WRN overexpression or SMARCA5 knock-down adipocytes. (N = 3 biological replicates). **j**, **k** qRT-PCR analysis of *pparγ* and *cebpα* at 2 dpf, 4 dpf, and 14 dpf between *wrn*^*−/−*^ mutant zebrafish and ASOs treated zebrafish (N = 3 biological replicates). **l**, **m** qRT-PCR analysis of *per1a* and *rorb* at 2 dpf, 4 dpf, and 14 dpf between *wrn*^*−/−*^ mutant zebrafish and ASOs treated zebrafish (N = 3 biological replicates). **n** Illustration of three different regions in human SMARCA5 promoters selected for dual-luciferase assay. **o** Dual-luciferase assay analysis of SMARCA5 transcription activity (N = 3 biological replicates). Data are presented as the mean ± S.D. Statistical analysis was performed using two-tailed unpaired Student’s t-test. *P < 0.05, **P < 0.01, ***P < 0.001

Based on the above findings, we firstly examined the expression of *SMARCA5* during early-stage adipogenesis. Loss of *WRN* led to higher expression of *SMARCA5* in WRN KO adipocytes when compared with the expression in WT cells, as shown in Fig. 6a, b. Additionally, ectopic overexpression of *WRN* in the WRN KO adipocytes mitigated this dysregulation (Fig. 6a, b). Similar results were obtained in the *wrn*^*−/−*^ mutant zebrafish. S*marca5* was abundantly expressed in *wrn*^*−/−*^ mutant zebrafish at 4 and 14 dpf (Fig. 6c). We microinjected human *WRN* mRNA at the one-cell stage of zebrafish embryos and noted that overexpression of *WRN* normalized *smarca5* expression (Fig. 6c). These data indicate that WRN regulates *SMARCA5* expression.

We next examined the relationship between WRN and SMARCA5 in adipogenesis. We either ectopically overexpressed *WRN* or knocked down *SMARCA5* using lentivirus and collected the samples on day 5 of adipogenesis. Overexpression of *WRN* or knockdown of *SMARCA5* normalized adipogenesis compared to that of WRN KO adipocytes as results Fig. 6d–g showed. Similarly, overexpression of WRN or SMARCA5 restored the expression of the early-stage marker *PER1* (Fig. 6h) and late-stage marker *RORB* (Fig. 6i). Additionally, we designed two antisense oligonucleotides (ASOs) targeting *smarca5* to knock it down in zebrafish. The *smarca5* knockdown efficiency was firstly examined as shown in Additional file 2: Fig. S6. Next, we microinjected two ASOs into zebrafish embryos and checked the expression of representative adipocyte-associated genes. As shown in Fig. 6j–m, the mRNA expression of *pparγ* (j), *cebpα* (k), *per1a* (l), and *rorb* (m) was restored in the ASOs group of *wrn*^*−/−*^ mutant zebrafish. Next, we selected three different regions in the *SMARCA5* promoter: region 1 from − 1500 to − 800 position, region 2 from − 850 to − 200 position, and region 3 from − 250 to + 150 position for further analysis (Fig. 6n). We cloned these regions into luciferase plasmids and transfected them into WRN knock down HEK293T cells, and observed an enhanced SMARCA5 luciferase activity upon WRN KD (Fig. 6o). Taken together, these data suggest that the aberrant upregulation of *SMARCA5* is at least one of the main causes of accelerated adipogenesis in WS.

### SIRT1 regulates SMARCA5 expression during adipogenesis in hMSCs and zebrafish models

**Fig. 7:**
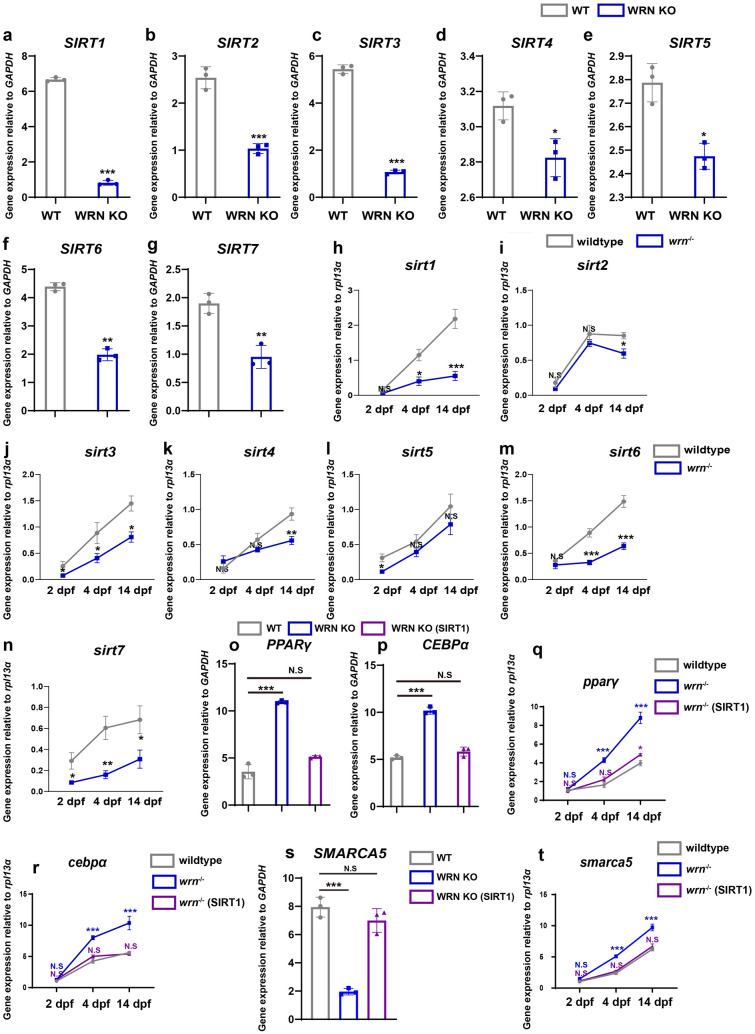
SIRT1 regulates SMARCA5 expression during adipogenesis in hMSCs and zebrafish models. **a**–**g** qRT-PCR analysis of SIRT family (SIRT1-7) on day 5 during adipogenesis (N = 3 biological replicates). **h**–**n** qRT-PCR analysis of sirt1-7 at 2 dpf, 4 dpf, and 14 dpf in zebrafish (N = 3 biological replicates). **o**, **p** qRT-PCR analysis of *PPARγ* (**o**) and *CEBPα* (**p**) among WT, WRN KO, and WRN KO (SIRT1) on day 5 during adipogenesis (N = 3 biological replicates). **q**, **r** qRT-PCR analysis of *pparγ* (**q**) and *cebpα* (**r**) among wildtype, *wrn*^*−/−*^ mutant zebrafish, *wrn*^*−/−*^ mutant zebrafish (SIRT1) (N = 3 biological replicates). **s** qRT-PCR analysis of *SMARCA5* among WT, WRN KO, and WRN KO (SIRT1) on day 5 during adipogenesis (N = 3 biological replicates). **t** qRT-PCR analysis of *smarca5* among wildtype, *wrn*^*−/−*^ mutant zebrafish, *wrn*^*−/−*^ mutant zebrafish (SIRT1) (N = 3 biological replicates). Data are presented as the mean ± S.D. Statistical analysis was performed using two-tailed unpaired Student’s t-test. *P < 0.05, **P < 0.01, ***P < 0.001

Next, we are interested to figure out the underlying mechanism explaining why the loss of WRN leads to transient upregulation of *SMARCA5* during adipogenesis. Based on our omics results presented in Fig. 4, we noted that NAD^+^ biosynthesis activity declined in the WRN KO adipocytes. NAD^+^ levels influence the activity of the mammalian sirtuin (SIRT) proteins [41, 42], which are deacetylases that participate in gene silencing, extension of life, and cellular metabolism/mitochondrial function among others [43, 44]. Das et al. reported that reduced blood flow with aging was associated with reduced NAD^+^ levels and SIRT1 activity. Treatment with NMN (an NAD^+^ intermediate) activated the NAD^+^-SIRT1 axis and promoted vascular endothelial cells growth, and stimulates sprouting angiogenesis [45]. Zhao et al. demonstrated that NAD^+^ ameliorated cognitive impairment and impaired neuroinflammation in chronic cerebral hypoperfusion (CHH) models in vivo and in vitro. Mechanistically, NAD^+^ administration activated the Sirt1/PGC-1 pathway, indicating an important role for the NAD^+^/Sirt1 pathway in neuroprotection [46]. Therefore, we speculated whether SMARCA5 activity is regulated by SIRT family. To test our hypothesis, we first checked the mRNA expression level of *SIRT1-7* on day 5 of adipocyte development in hMSCs. As Fig. 7a–g shown, *SIRT1*, *2*, *3*, and *6* showed a significantly decreased expression in WRN KO adipocytes compared to WT cells. In addition, we examined the expression of *sirt1-7* in zebrafish (Fig. 7h–n). Similarly, we noted that sirt members (*sirt1*, *3*, *6*) decreased in *wrn*^*−/−*^ mutant zebrafish. It has previously been reported that SIRT1 was crucial for adipogenesis, and overexpression of SIRT1 inhibited adipogenesis in 3T3-L1 cells [47]. Similarly, we overexpressed SIRT1 in WRN KO adipocytes and checked the expression of *PPARγ* and *CEBPα* on day 5 during adipogenesis. We found that overexpression of SIRT1 rescued the expression of *PPARγ* and *CEBPα* (Fig. 7o, p). Similar results were observed in zebrafish models as well (Fig. 7q, r). Additionally, the expression of *SMARCA5* came back to normal again both in vitro and in vivo (Fig. 7s, t). Based on the above results, we demonstrated that SIRT1 could regulate SMARCA5 expression during adipogenesis in WS.

### Nicotinamide riboside (NR) rescues metabolic dysfunction in WRN KO adipocytes

NAD^+^ metabolism is essential for sirtuin activity, and our omics data revealed that NAD^+^ metabolism pathway is dysregulated in WRN KO cells (Fig. 4). It has previously been shown that NAD^+^ levels declined in both plasma and primary fibroblasts from WS patients [48] and animal models [20], and that NR treatment inhibited the accelerated aging features, and restored lifespan and health span in both WS and other age-related disease [49]. We therefore decided to examine the effects of NR treatment on the adipocyte metabolic abnormalities seen in WRN KO cells and *wrn*^*−/−*^ mutant zebrafish.

**Fig. 8:**
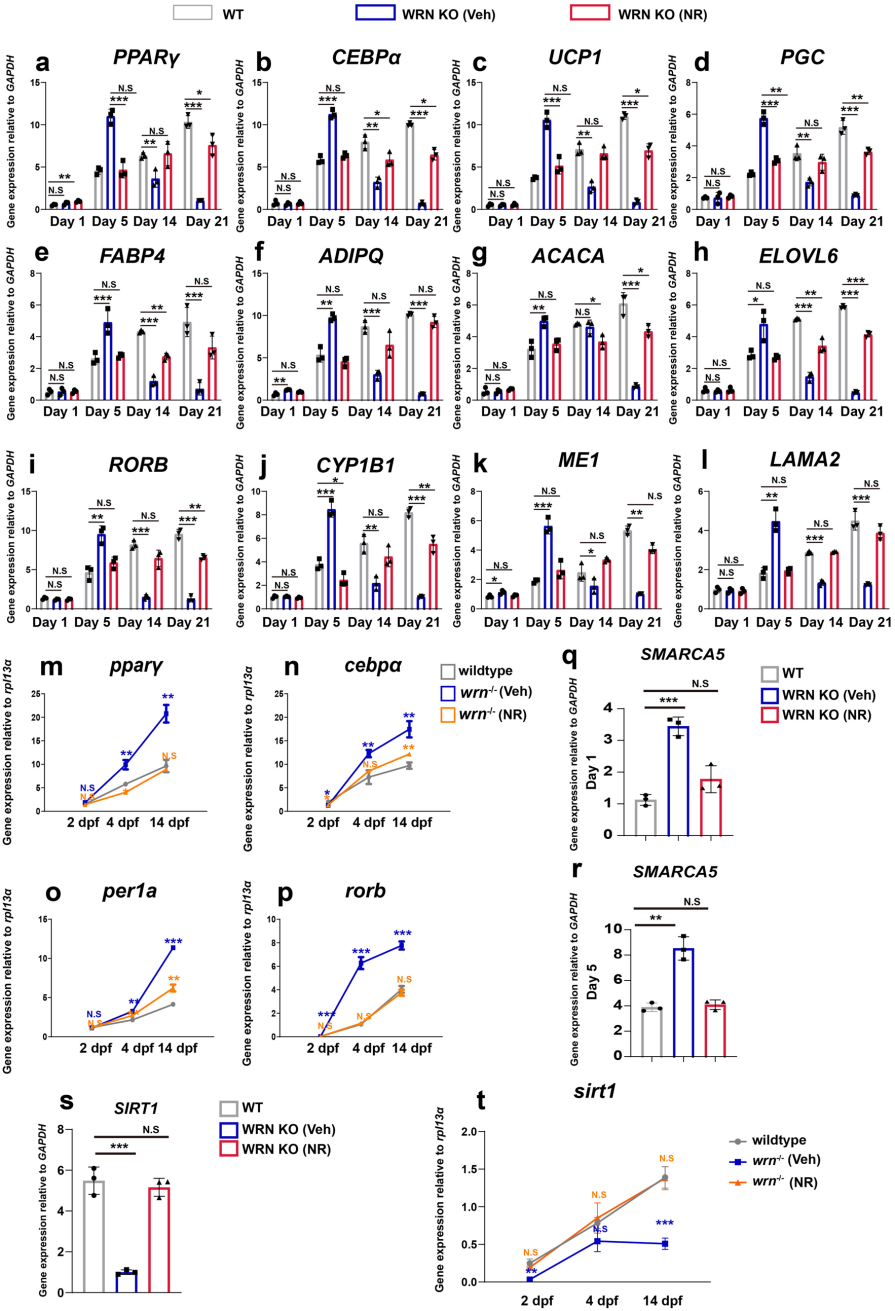
Nicotinamide riboside (NR) normalizes metabolism in WRN KO adipocytes. **a**–**h** qTR-PCR analysis of selected adipogenic genes *PPARγ* (**a**), *CEBPα* (**b**), *UCP1* (**c**), *PGC* (**d**), *FABP4* (**e**), *ADIPQ* (**f**), *ACACA* (**g**), and *ELOVL 6* (**h**) among WT, WRN KO (veh), and WRN KO (NR) in hMSCs (N = 3 biological replicates). **i**–**l** qTR-PCR analysis of selected adipogenic circadian genes *RORB* (**i**), *CYP1B1* (**j**), *ME1* (**k**), and *LAMA2* (**l**) among WT, WRN KO (veh), and WRN KO (NR) (N = 3 biological replicates). **m**–**p** qTR-PCR analysis of selected adipogenic circadian genes *pparγ* (**m**), *cebpα* (**n**), *per1a* (**o**), and *rorb* (**p**) among wildtype, *wrn*^*−/−*^ mutant zebrafish (veh), and *wrn*^*−/−*^ mutant zebrafish (NR) (N = 3 biological replicates). **q**, **r** qTR-PCR analysis of *SMARCA5* on day 1 (**q**) and day 5 (**r**) during adipogenesis (N = 3 biological replicates). **s** qTR-PCR analysis of *SIRT1* on day 5 during adipogenesis (N = 3 biological replicates). **t** qTR-PCR analysis of *sirt1* at 2 dpf, 4 dpf, and 14 dpf in zebrafish (N = 3 biological replicates). Data are presented as the mean ± S.D. Statistical analysis was performed using two-tailed unpaired Student’s t-test. *P < 0.05, **P < 0.01, ***P < 0.001

NAD^+^ levels were measured in WT and WRN KO adipocytes during adipogenesis, and showed decreased NAD^+^ levels in WRN KO adipocytes compared to WT cells (Additional file 2: Fig. S7). Next, we treated the WRN KO adipocytes with NR and the expression level of representative genes expressed in adipocytes (*PPARγ* (a), *CEBPα* (b), *UCP1* (c), *PGC* (d), *FABP4* (e), *ADIPQ* (f), *ACACA* (g), and *ELOVL6* (h)) were checked. The results showed that NR treatment in WRN KO adipocytes normalized the expression of adipocyte-related genes compared to WRN vehicle treated cells (Fig. 8a–h). Additionally, we validated the effect of NR in the WS-hESC model (Additional file 2: Fig. S8a–d) and showed that NR also restoreed white and brown adipocyte differentiation.

We next evaluated the expression of late adipogenic circadian genes (Fig. 8i–l). The expressions of *RORB* (i), *CYP1B1* (j), *ME1* (k), and *MALA2* (l) were restored by NR supplementation on day 5 of adipogenesis in WRN KO adipocytes. In line with our in vitro findings, adipocyte development was also normalized in *wrn*^*−/−*^ zebrafish after NR treatment (Fig. 8m–p).

Finally, we investigated whether NR could mitigate the dysregulation of *SMARCA5*. To this end, we checked the mRNA expression of *SMARCA5* with or without NR supplementation during adipogenesis. As Fig. 8q, r showed, NR normalized SMARCA5 expression on days 1 and day 5 of adipogenesis. Additionally, the expression of *SIRT1* and *sirt1* both increased after NR treatment (Fig. 8s, t). Collectively, our results indicate that NR supplementation has a promising effect in the treatment of abnormal adipocyte metabolism in WS.

## Discussion

Besides progeria, WS is also accompanied with metabolic dysregulation. Our study shows that loss of WRN causeed transient upregulated adipogenesis at an early stage, both in vitro and in vivo. In parallel, the adipogenic activities declined, which is in agreement with other study [50]. In this study, we also used hESCs and differentiated them into white and brown adipocytes separately [25] and observed that the effect of WRN on these two types of adipocytes was similar and did not show any difference. Recent work has shown that over-nutrition induced adipose progenitor cells (APCs) senescence exhibited transient activation of browning adipocytes and UCP1 expression in a prematurity model in which the tissue-specific telomerase reverse transcriptase (TERT) gene was knocked out in mice [51]. Similarly, another study reported that *Zmpste24*-null progeroid mice (*Zmpste24*^*−/−*^), which showed nuclear lamina defects and accumulated unprocessed prelamin A, exhibited higher levels of adipogenesis and increased mRNA expression of *C/EBPα* and *PPARγ* in the bone marrow of *Zmpste24*^*−/−*^ mice, indicating that aging might promote adipogenesis and inhibit osteogenesis [52]. Furthermore, we checked the early and late stages of adipocyte-specific differentiation genes separately. It has been reported that RORB was up-regulated only on days 14 and 21, which coincided with the maturation of adipocytes and probably could be an indicator of the mature of adipocyte [30]. Here we observed upregulation of RORB on day 5 in WRN KO adipocytes comparted to that in WT cells, indicating the premature of adipocytes in WS. Our data also present an accelerated adipogenesis in *wrn*^*−/−*^ mutant zebrafish model at an early stage. Interestingly, it has been shown that *Caenorhabditis elegans* worms deficient in *wrn* exhibited more fat and lipid accumulation with age [48], which is consistent with the results of our study.

Our RNA-seq data revealed that chromatin remodeling and chromosome rearrangement were upregulated in WRN adipocytes, suggesting that dynamic chromatin alterations might be one of the regulatory mechanisms. Chromatin accessibility is a crucial factor regulating gene expression [53]. Regulatory elements selectively bind to accessible chromatin regions, which is important for gene transcription and expression [54]. It has been noted that chromatin accessibility showed dynamic changes during adipocyte differentiation [55]. Enhanced chromatin accessibility was noted in adipose tissue with increased lipid accumulation from normal-weight women with polycystic ovary tissue (PCOS), suggesting a correlation between chromatin accessibility and gene expression [56]. ATAC-seq analysis showed increased chromatin accessibility in WRN adipocytes at an early stage of differentiation. Moreover, RPGC analysis showed adipocyte-related genes were in a relatively more open states, indicating a possible mechanism for accelerated adipogenesis in WS. Aging can also change chromatin accessibility [57, 58]. Aging in murine liver led to elevated chromatin accessibility at promoter regions but did not enhance transcriptional output [58]. Additionally, aging accelerated the rate of elongation by RNA polymerase II (Pol II), which led to more gene transcription [58].

To determine how the loss of WRN caused aberrant chromatin accessibility, ChIP-seq was performed. We noted that the expression of the chromatin remodeler, *SMARCA5* was upregulated during the early stage of adipogenesis. SMARCA5 belongs to the SWI/SNF family and is responsible for chromatin remodeling [59]. It has been shown that SMARCA5 interacted with nuclear proteins to facilitate chromatin remodeling and gene expression in hematopoietic stem and progenitor cells (HSPCs) [60]. Previous work has indicated that activation of the *PPARγ2* gene occurred only when followed by subsequent interacting with SWI/SNF enzymes and TFIIH factors [13]. Here, our data showed that rescue with WRN could normalize *SMARCA5* expression in WRN KO adipocytes, and knock-down the expression of *SMARCA5* restored adipogenesis in WS. Taken together, these findings suggest a potential mechanism by which WRN works with SMARCA5 in regulating adipogenesis in WS.

Decreased levels of NAD^+^ is observed with aging and is suggested to be a major factor in the progression of cellular dysfunction and age-related diseases [22]. Therefore, regulation of NAD^+^ levels offer a promising therapeutic alternative for various aging-related diseases, either through genetic approaches or pharmacological supplementation [20, 22]. Boosting NAD^+^ levels by supplementing with NR and/or NMN have previously been shown to activate SIRT1 and maintain mitochondrial and metabolic functions, which further prolonged the longevity of mice [61, 62]. Our data demonstrated that NR supplementation normalizes adipogenesis, and moreover that NR restores the expression of late-stage adipocyte-specific genes. NAD^+^ mediates genome stability and chromatin complexity [22, 63]. For example, diphtheria toxin-like ADP-ribosyltransferases (ARTDs, also named as poly-ADP-ribose polymerases (PARPs)) are major consumers of cellular NAD^+^. Its family member PARP1, which regulates PARylation of histone tails, leads to recruitment of chromatin remodeling proteins such as SMARCA5, promoting DNA repair and genome stability [64]. Our data showed that supplementation of NR normalized *SMARCA5* expression during early adipocyte differentiation. However, the mechanisms linking NR and SMARCA5 require further investigation.

## Conclusions

Here, we demonstrated the linkage between the chromatin stabilizing protein WRN and disruptions in fat-metabolism and adipogenesis, by using of both stem cells and zebrafish model of WS. Our results reveal a new mechanism of WRN’s influence on the early stages of adipogenesis and suggest a treatment for metabolic dysfunction in WS. They may also have relevance to therapeutics for aging and age-related diseases.

### Supplementary Information

**Additional file 1.** All the primer sequences used in the experiments.

**Additional file 2: Figure S1.** Generation of WRN knock out (WRN KO) hMSCs. a. Cell sorting by the flow cytometry method. Representative data was shown (N = 3 biological replicates). b. *WRN* knock out efficiency was examined by qRT-PCR (N = 3 biological replicates). Data are presented as the mean ± S.D. Statistical analysis was performed using two-tailed unpaired Student’s t-test. *P < 0.05, **P < 0.01, ***P < 0.001. **Figure S2.** RNA-seq quality control examination. a. Mean quality scores of each RNA-seq samples. b. Per sequence quality scores of each RNA-seq samples. c. Pearson’s correlation between biological replicates. **Figure S3.** ATAC-seq quality control examination. a. Mean quality scores of each ATAC-seq samples. b. Per sequence quality scores of each ATAC-seq samples. **Figure S4.** ChIP-seq quality control examination. a. Mean quality scores of each ATAC-seq samples. b. Per sequence quality scores of each ChIP-seq samples. **Figure S5.** Multiple omics analysis during adipogenesis. a–d. Integrative analysis RNA-seq, ChIP-seq, and ATAC-seq on day 1 and 5 during adipogenesis between the WT and WRN KO adipocytes. e, f. Heatmap analysis of adipogenesis on day 1 and 5. **Figure S6.** Examination of ASOs efficiency. qRT-PCR analysis of two ASOs in zebrafish (N = 3 biological replicates). Data are presented as the mean ± S.D. Statistical analysis was performed using two-tailed unpaired Student’s t-test. *P < 0.05, **P < 0.01, ***P < 0.001. **Figure S7.** NAD+/NADH ration declines in WRN adipocytes. a. NAD+/NADH ration levels between WT and WRN adipocytes (N = 3 biological replicates). Data are presented as the mean ± S.D. Statistical analysis was performed using two-tailed unpaired Student’s t-test. *P < 0.05, **P < 0.01, ***P < 0.001. **Figure S8.** NR restores while and brown adipocytes. a–d. qRT-PCR analysis of *PPARγ*,*CEBPα*, *CIDEA*, and *UCP1*expression (N = 3 biological replicates). Data are presented as the mean ± S.D. Statistical analysis was performed using two-tailed unpaired Student’s t-test. *P < 0.05, **P < 0.01, ***P < 0.001.

## Data Availability

The sequencing data were uploaded under the accession number: GSE248012. All data generated or analyzed during this study are available from the corresponding author on reasonable request.

## Abbreviations

WS: Werner syndrome
ChIP-seq: Chromatin immunoprecipitation sequencing
ATAC-seq: Assay for Transposase-Accessible Chromatin using sequencing
SIRT: Sirtuin
NAD^+^: Nicotinamide adenine dinucleotide
NR: Nicotinamide riboside
EB: Embryonic body
