# Supplementary material for: WRN loss accelerates abnormal adipocyte metabolism in Werner syndrome
Source: Cell Biosci. 2024 Jan 6;14:7. doi: 10.1186/s13578-023-01183-4 (PMC10770995; doi:10.1186/s13578-023-01183-4)
Supplement: Supplementary file 2 — Additional file 2: Figure S1. Generation of WRN knock out (WRN KO) hMSCs. a. Cell sorting by the flow cytometry method. Representative data was shown (N = 3 biological replicates). b. WRN knock out efficiency was examined by qRT-PCR (N = 3 biological replicates). Data are presented as the mean ± S.D. Statistical analysis was performed using two-tailed unpaired Student’s t-test. *P < 0.05, **P < 0.01, ***P < 0.001. Figure S2. RNA-seq quality control examination. a. Mean quality scores of each RNA-seq samples. b. Per sequence quality scores of each RNA-seq samples. c. Pearson’s correlation between biological replicates. Figure S3. ATAC-seq quality control examination. a. Mean quality scores of each ATAC-seq samples. b. Per sequence quality scores of each ATAC-seq samples. Figure S4. ChIP-seq quality control examination. a. Mean quality scores of each ATAC-seq samples. b. Per sequence quality scores of each ChIP-seq samples. Figure S5. Multiple omics analysis during adipogenesis. a–d. Integrative analysis RNA-seq, ChIP-seq, and ATAC-seq on day 1 and 5 during adipogenesis between the WT and WRN KO adipocytes. e, f. Heatmap analysis of adipogenesis on day 1 and 5. Figure S6. Examination of ASOs efficiency. qRT-PCR analysis of two ASOs in zebrafish (N = 3 biological replicates). Data are presented as the mean ± S.D. Statistical analysis was performed using two-tailed unpaired Student’s t-test. *P < 0.05, **P < 0.01, ***P < 0.001. Figure S7. NAD+/NADH ration declines in WRN adipocytes. a. NAD+/NADH ration levels between WT and WRN adipocytes (N = 3 biological replicates). Data are presented as the mean ± S.D. Statistical analysis was performed using two-tailed unpaired Student’s t-test. *P < 0.05, **P < 0.01, ***P < 0.001. Figure S8. NR restores while and brown adipocytes. a–d. qRT-PCR analysis of PPARγ,CEBPα, CIDEA, and UCP1expression (N = 3 biological replicates). Data are presented as the mean ± S.D. Statistical analysis was performed using two-tailed unpaired Student’s [file 13578_2023_1183_MOESM2_ESM.docx]

WRN loss accelerates abnormal adipocyte metabolism in Werner Syndrome

Yuyao Tian^1,2^, Sofie Lautrup^3^, Patrick Wai Nok Law^1^, Ngoc-Duy Dinh^2^, Evandro Fei Fang^3^, Wai-Yee Chan^1,4,5,6*^

^1^ School of Biomedical Sciences, Faculty of Medicine, the Chinese University of Hong Kong, Shatin, N.T., Hong Kong SAR.

^2^ Department of Biomedical Engineering, the Chinese University of Hong Kong, Shatin, N.T., Hong Kong SAR

^3^ Department of Clinical Molecular Biology, University of Oslo and Akershus University Hospital, 1478 Lørenskog, Norway

^4^ Hong Kong Branch CAS Center of Excellence for Animal Evolution and Genetics, the Chinese University of Hong Kong, Shatin, N.T., Hong Kong SAR.

^5^ CUHK-SDU University Joint Laboratory on Reproductive Genetics, the Chinese University of Hong Kong, Shatin, N.T., Hong Kong SAR.

^6^ MOE Key Laboratory of Regenerative Medicine (CUHK-Jinan University), the Chinese University of Hong Kong, Shatin, N.T., Hong Kong SAR.


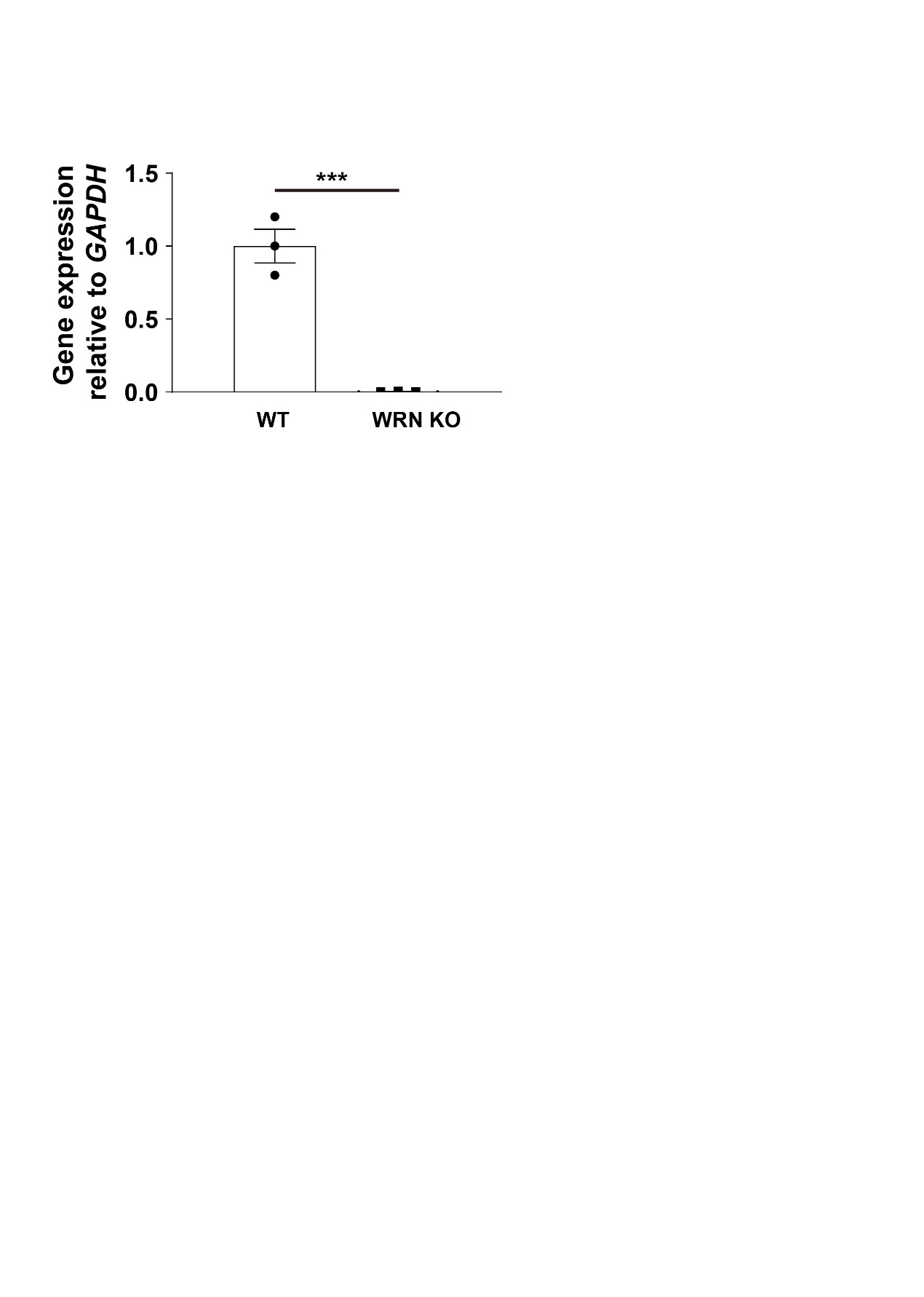


Additional File 2 Fig. 1 Generation of WRN knock out (WRN KO) hMSCs. *WRN* knock out efficiency was examined by qRT-PCR (N=3 biological replicates). Data are presented as the mean ± S.D. Statistical analysis was performed using two-tailed unpaired Student’s t-test. **P* < 0.05, ***P* < 0.01, ****P* < 0.001.


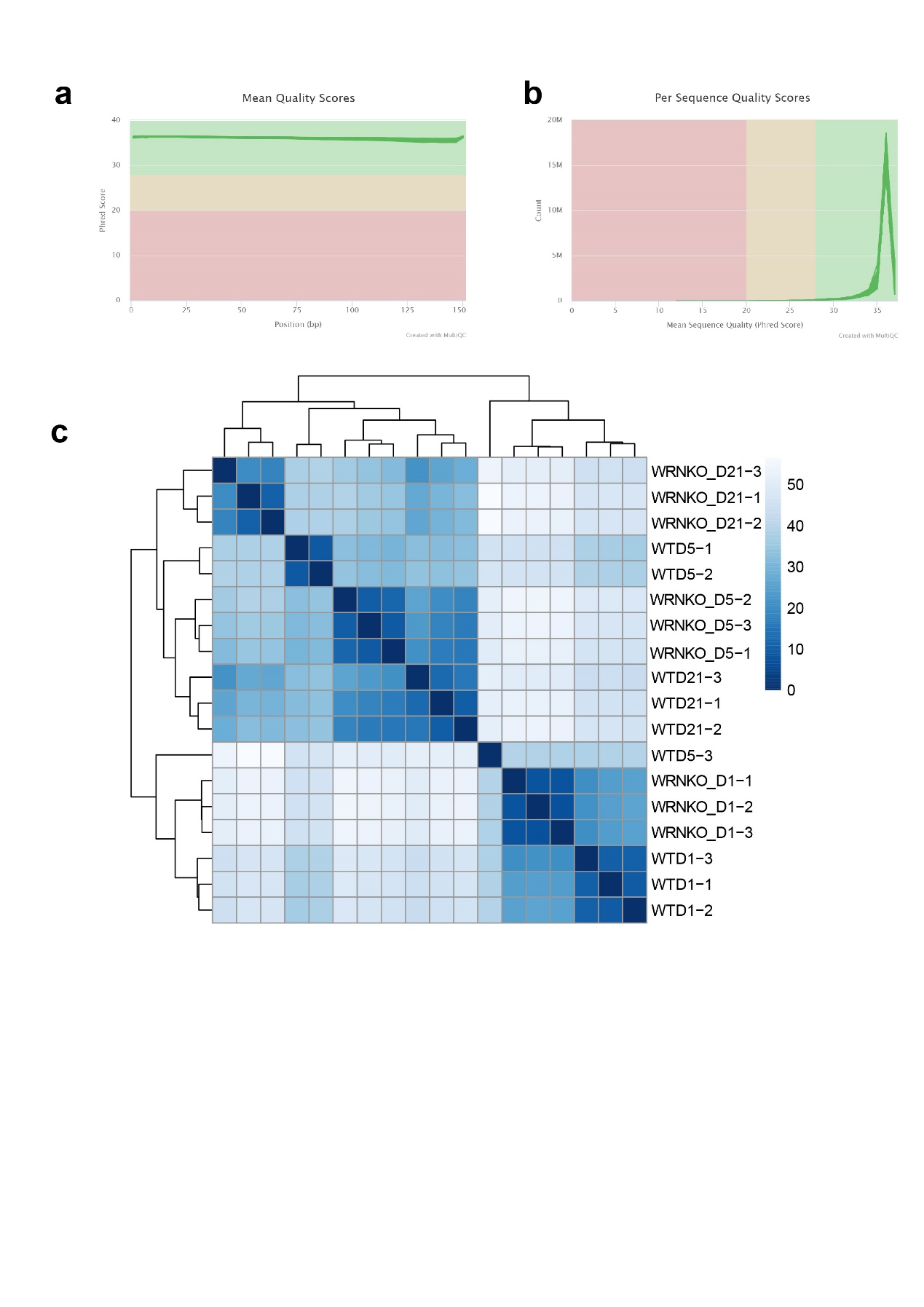


Additional File 2 Fig. 2 RNA-seq quality control examination. a. Mean quality scores of each RNA-seq samples. b. Per sequence quality scores of each RNA-seq samples. c. Pearson’s correlation between biological replicates.


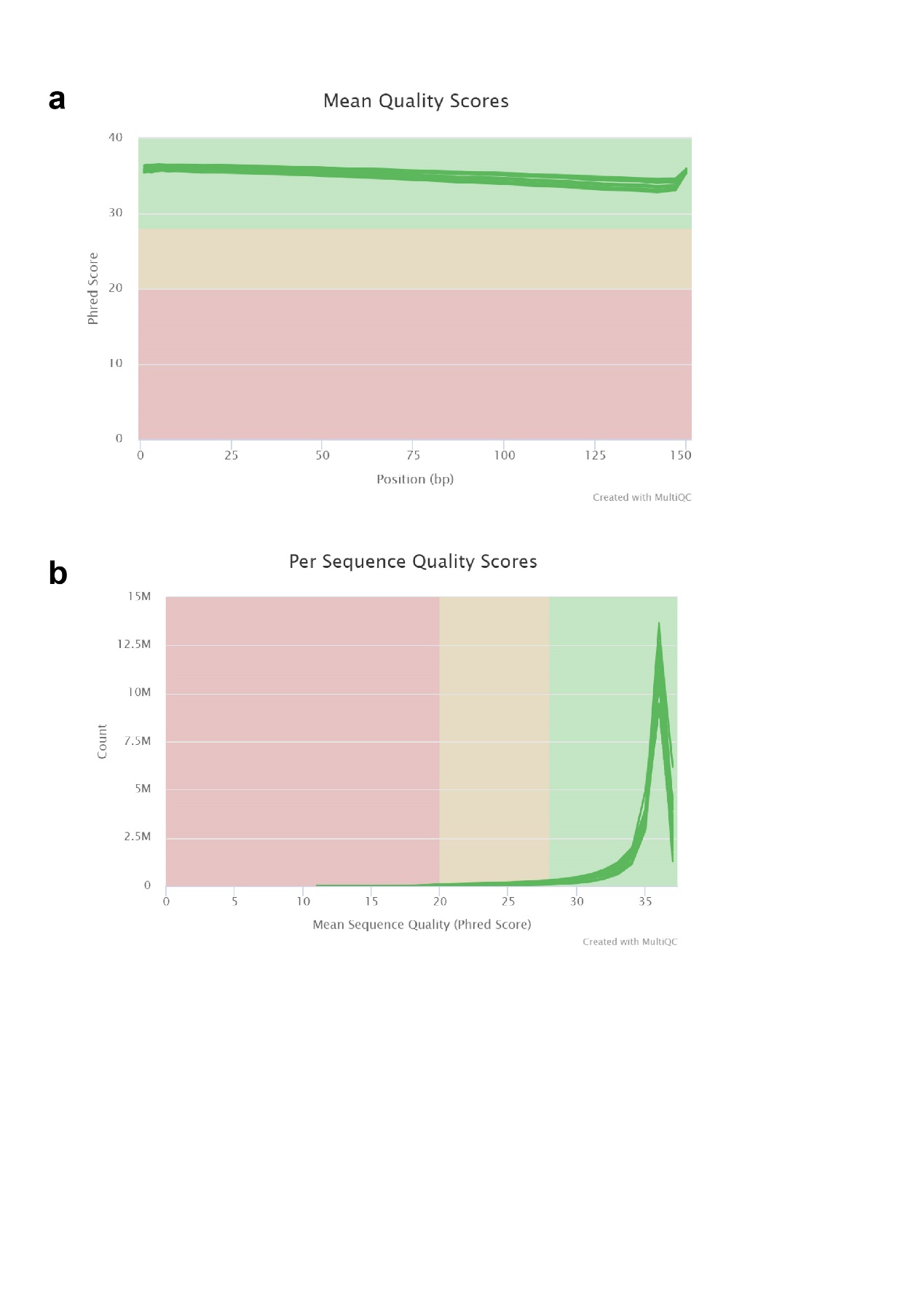


Additional File 2 Fig. 3 ATAC-seq quality control examination. a. Mean quality scores of each ATAC-seq samples. b. Per sequence quality scores of each ATAC-seq samples.


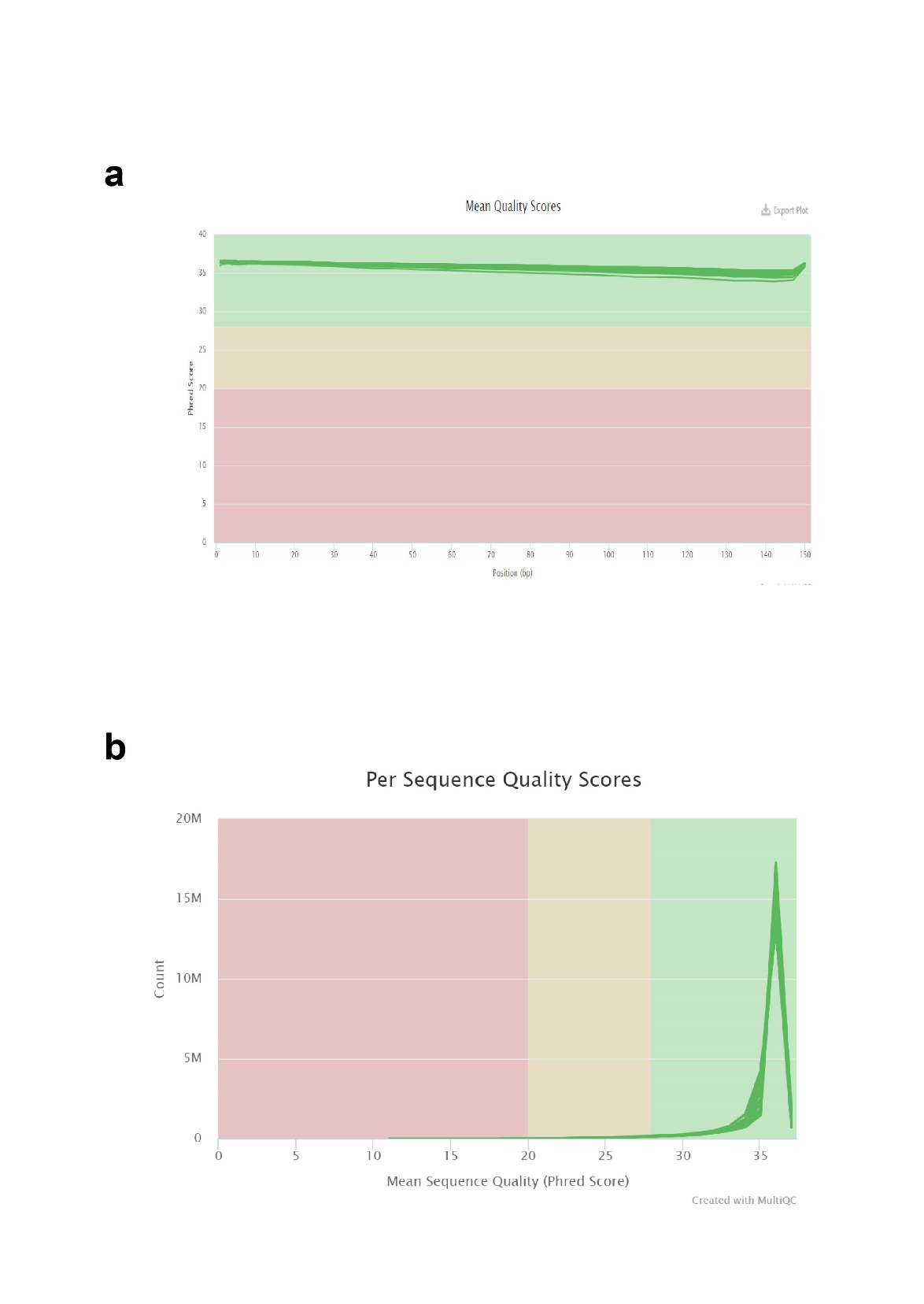


Additional File 2 Fig. 4 ChIP-seq quality control examination. a. Mean quality scores of each ATAC-seq samples. b. Per sequence quality scores of each ChIP-seq samples.


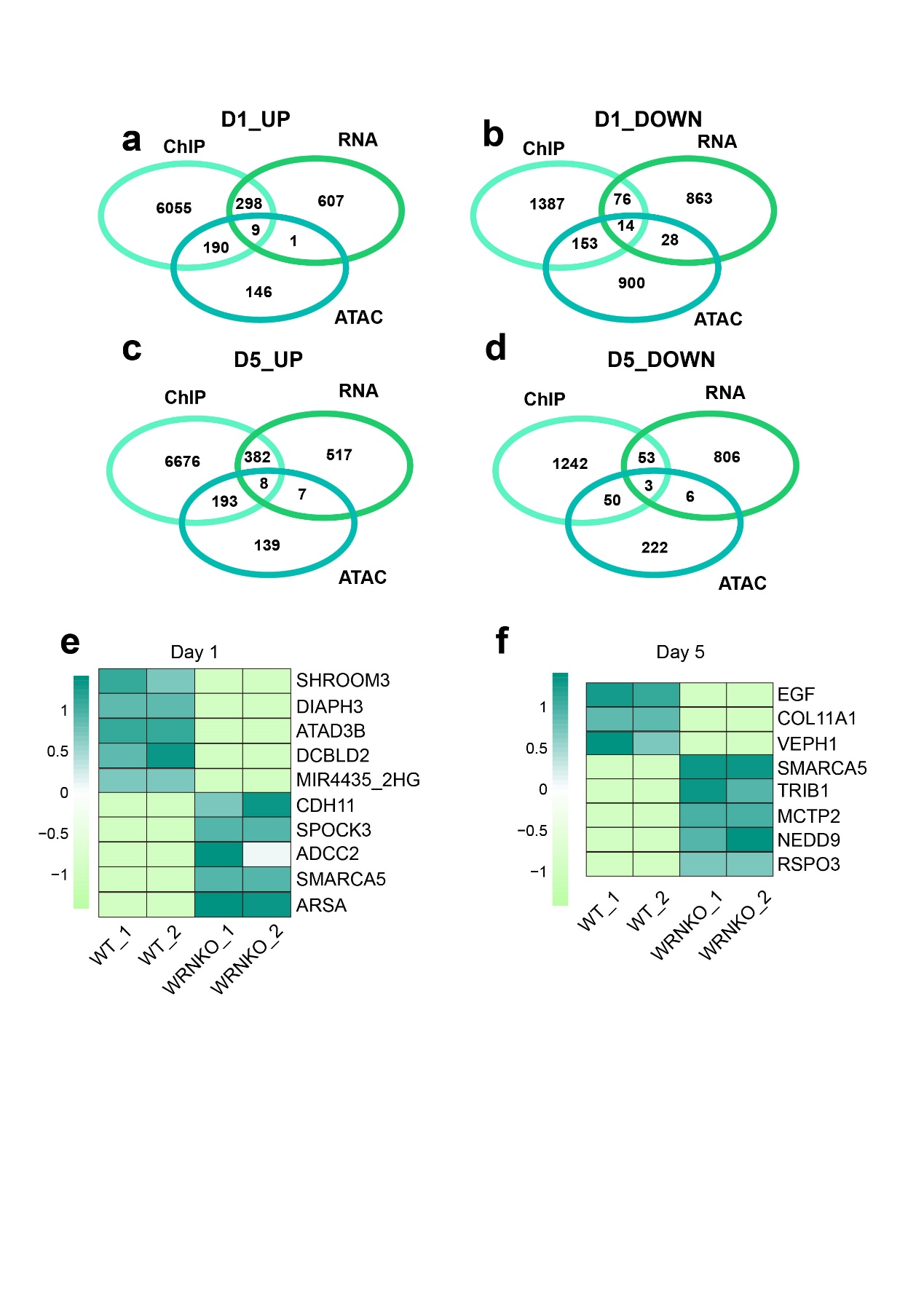


Additional File 2 Fig. 5 Multiple omics analysis during adipogenesis. a-d. Integrative analysis RNA-seq, ChIP-seq, and ATAC-seq on day 1 and 5 during adipogenesis between the WT and WRN KO adipocytes. e-f. Heatmap analysis of adipogenesis on day 1 and 5.


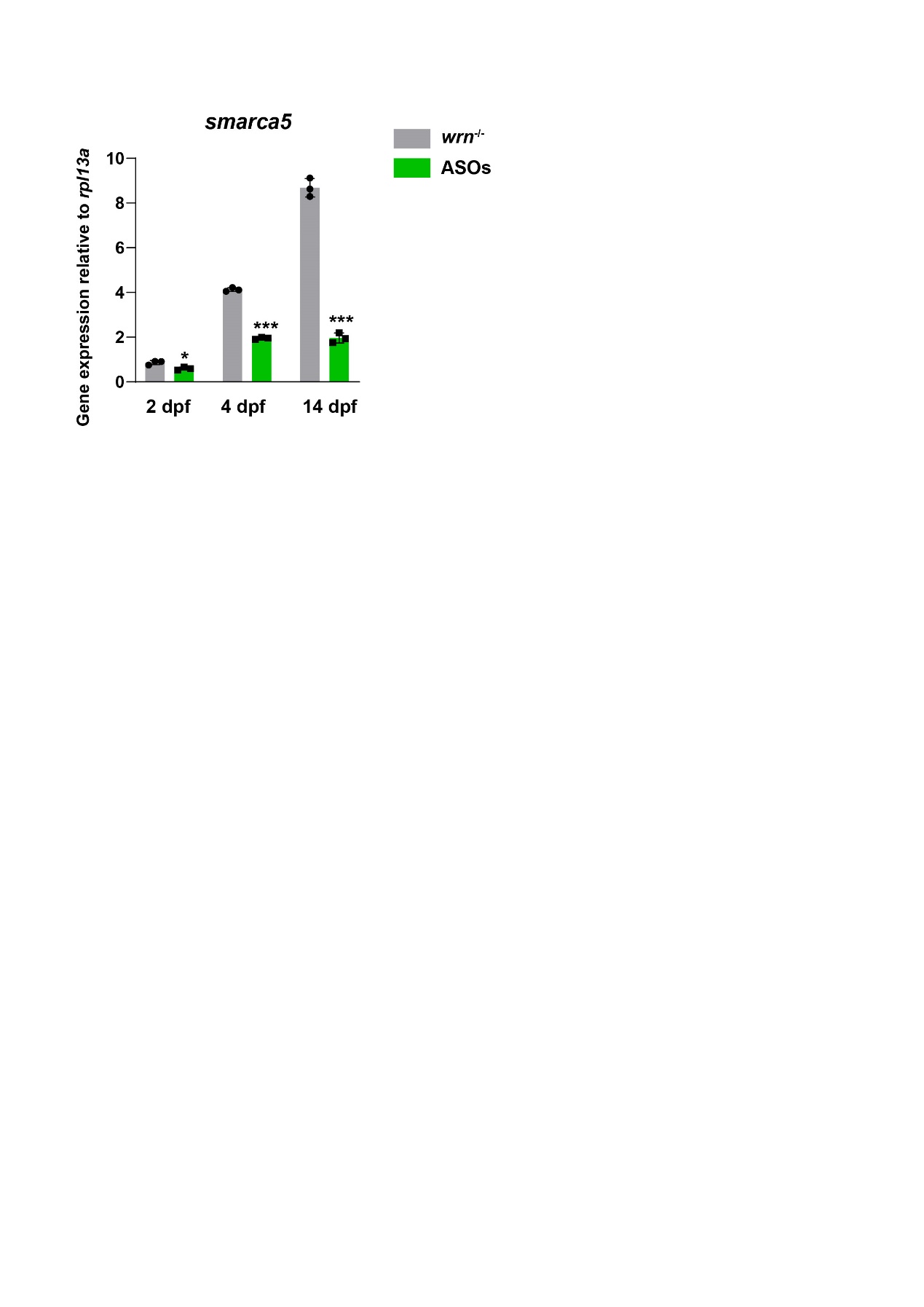


Additional File 2 Fig. 6 Examination of ASOs efficiency. qRT-PCR analysis of two ASOs in zebrafish (N=3 biological replicates). Data are presented as the mean ± S.D. Statistical analysis was performed using two-tailed unpaired Student’s t-test. **P* < 0.05, ***P* < 0.01, ****P* < 0.001.


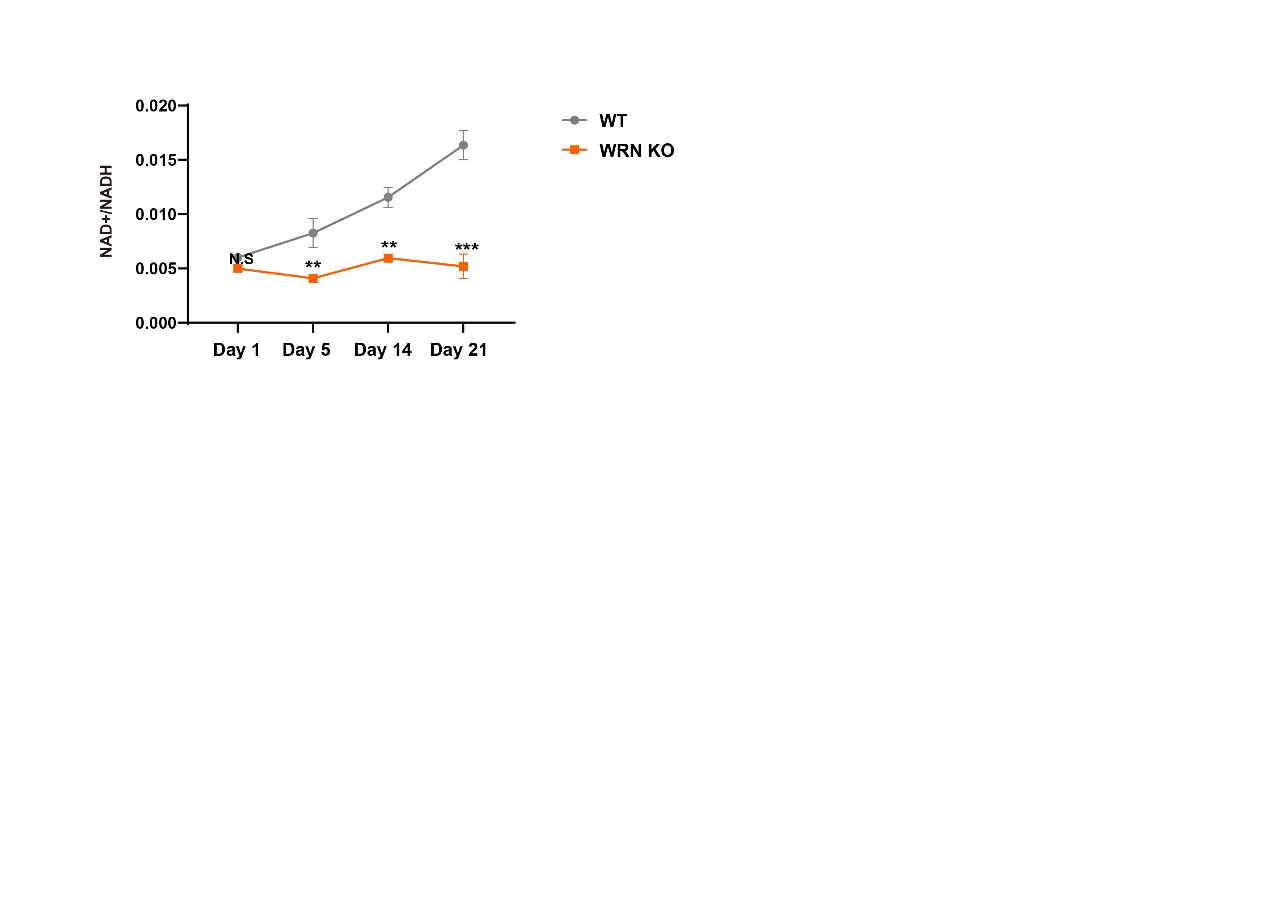


Additional File 2 Fig. 7 NAD^+^/NADH ration declines in WRN adipocytes. a. NAD^+^/NADH ration levels between WT and WRN adipocytes (N=3 biological replicates). Data are presented as the mean ± S.D. Statistical analysis was performed using two-tailed unpaired Student’s t-test. **P* < 0.05, ***P* < 0.01, ****P* < 0.001.


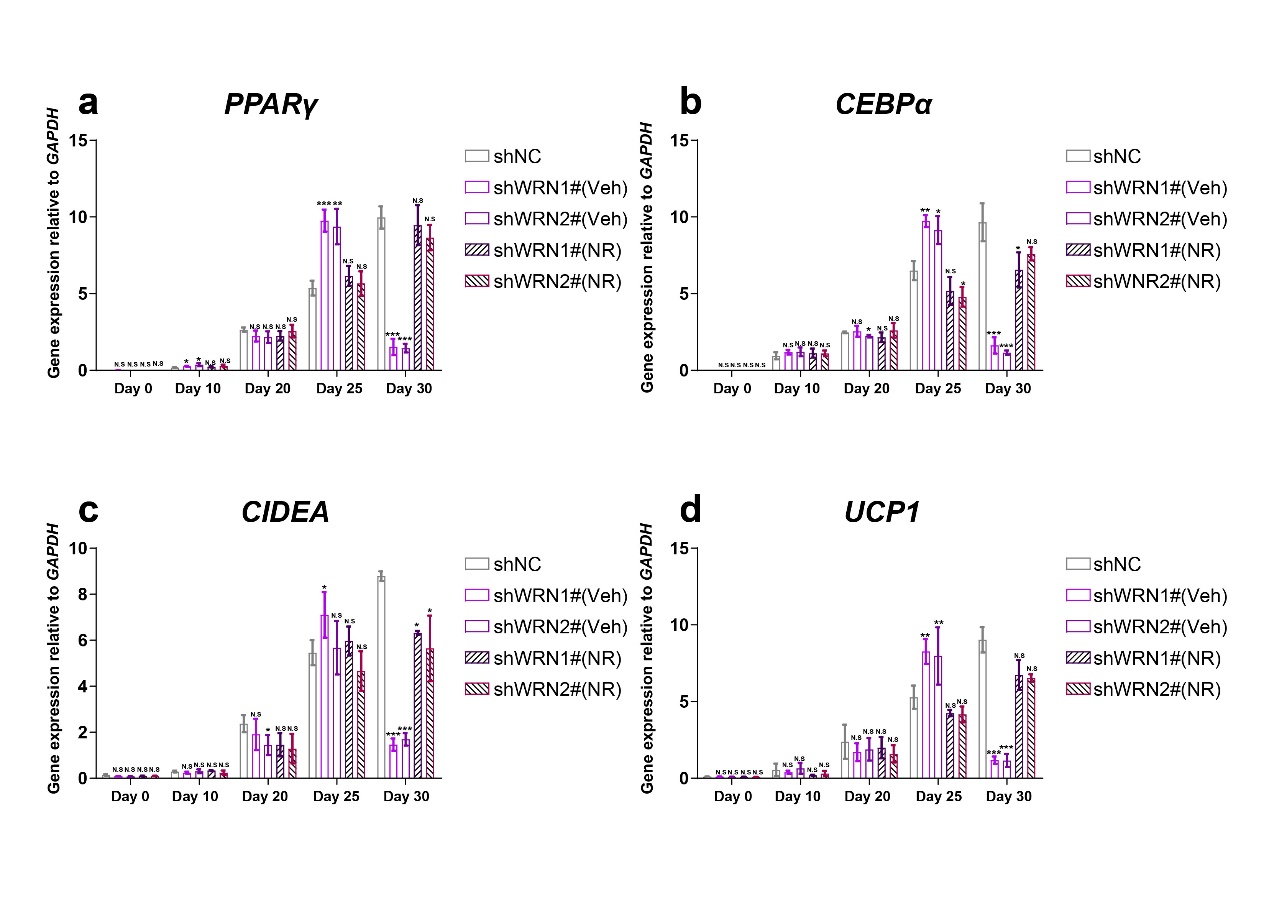


Additional File 2 Fig. 8 NR restores while and brown adipocytes. a-d. qRT-PCR analysis of *PPARγ*, *CEBPα*, *CIDEA*, and *UCP1* expression (N=3 biological replicates). Data are presented as the mean ± S.D. Statistical analysis was performed using two-tailed unpaired Student’s t-test. **P* < 0.05, ***P* < 0.01, ****P* < 0.001.
